# CT45A1‐mediated MLC2 (MYL9) phosphorylation promotes natural killer cell resistance and outer cell fate in a cell‐in‐cell structure, potentiating the progression of microsatellite instability‐high colorectal cancer

**DOI:** 10.1002/1878-0261.13736

**Published:** 2024-09-25

**Authors:** Hao‐Wei Teng, Hsiang‐Yueh Huang, Chun‐Chi Lin, Yuh‐Ching Twu, Wen‐Hao Yang, Wen‐Chun Lin, Hsin‐Yi Lan, Yen‐Yu Lin, Wei‐Lun Hwang

**Affiliations:** ^1^ Division of Medical Oncology, Department of Oncology Taipei Veterans General Hospital Taiwan; ^2^ School of Medicine National Yang Ming Chiao Tung University Taipei Taiwan; ^3^ Department of Biotechnology and Laboratory Science in Medicine National Yang Ming Chiao Tung University Taipei Taiwan; ^4^ Division of Colon and Rectum Surgery, Department of Surgery Taipei Veterans General Hospital Taiwan; ^5^ Department of Surgery, Faculty of Medicine, School of Medicine National Yang Ming Chiao Tung University Taipei Taiwan; ^6^ Graduate Institute of Biomedical Sciences, Research Center for Cancer Biology and Center for Molecular Medicine China Medical University Taichung Taiwan; ^7^ Department of Pathology, Fu Jen Catholic University Hospital Fu Jen Catholic University New Taipei City Taiwan; ^8^ School of Medicine, College of Medicine Fu Jen Catholic University New Taipei City Taiwan; ^9^ Cancer and Immunology Research Center National Yang Ming Chiao Tung University Taipei Taiwan

**Keywords:** cell‐in‐cell structure, colorectal cancer, CT45A1, microsatellite instability‐high, natural killer cells

## Abstract

Patients with microsatellite instability‐high (MSI‐H) colorectal cancer (CRC) have high tumor mutation burden and tumor immunogenicity, exhibiting a higher response rate to immunotherapy and better survival. However, a portion of MSI‐H CRC patients still experience adverse disease outcomes. We aimed to identify the tumor‐autonomous regulators determining these heterogeneous clinical outcomes. The Cancer Genome Atlas (TCGA) dataset was used to identify regulators in MSI‐H CRC patients with unfavorable outcomes. Stable CRC tumor clones expressing targeted regulators were established to evaluate migratory and stemness properties, immune cell vulnerability, and cell‐in‐cell (CIC) structure formation. RNA‐sequencing (RNA‐seq) was used to identify enriched biological pathways in stable CRC tumor clones. Clinicopathological characterization of formalin‐fixed paraffin‐embedded (FFPE) MSI‐H CRC specimens was performed to explore the underlying mechanisms involved. We showed that cancer/testis antigen family 45 member A1 (*CT45A1*) expression was upregulated in MSI‐H CRC patients with poor survival outcomes. CT45A1‐expressing microsatellite stable (MSS) CRC cells showed enhanced migratory ability. However, CT45A1‐expressing MSI‐H CRC cells, but not MSS CRC cells, showed higher resistance to natural killer (NK) cell cytotoxicity and served as outer cells in homotypic CIC structures, preventing exogenous or therapeutic antibody access to inner CRC cells. Inactivating RHO‐ROCK/MLCK‐MLC2 signaling with small‐molecule inhibitors or short‐hairpin RNAs (shRNAs) targeting myosin light chain kinase (*MYLK*) abolished NK cell resistance and reduced the outer cell fate of CT45A1‐expressing MSI‐H CRC cells. In MSI‐H CRC patients, CT45A1‐positive tumors exhibited increased MLC2 phosphorylation, increased outer cell fate, and decreased survival. We demonstrated that CT45A1 potentiates the advanced progression of MSI‐H CRC, and targeting MLC2 phosphorylation may enhance immunotherapy efficacy in CT45A1‐positive MSI‐H CRC patients.

## Graphical abstract

CT45A1, a vital driver of the RHO‐ROCK/MLCK‐MLC2 signaling, is found to enhance cancer cell resistance to natural killer (NK) cell killing and generate a protective cell‐in‐cell (CIC) structure in microsatellite instability‐high (MSI‐H) colorectal cancer (CRC) cells. This structure shields the inner CT45A1 (Low) cancer cells from targeted antibody therapy and potentially subsequent antitumor immunity, thereby driving aggressive MSI‐H CRC outcomes.

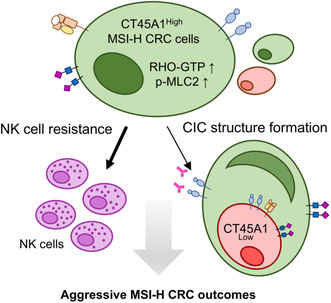

**Abbreviations**
ACD: antibody‐conjugated drugs
ACO: antibody‐conjugated oligonucleotides
ADCC: antibody‐dependent cellular cytotoxicity
AMPK: AMP‐activated protein kinase
BSA: bovine serum albumin
cDNA: complementary DNA
CIC: cell‐in‐cell
CIM: chronic idiopathic myelofibrosis
CMS: consensus molecular subtypes
CRC: colorectal cancer
CT45A1: cancer/testis antigen family 45 member A1
DAVID: database for annotation, visualization and integrated discovery
dMMR: DNA mismatch repair
DMSO: dimethyl sulfoxide
EDTA: ethylenediaminetetraacetic acid
EMT: epithelial–mesenchymal transition
EVs: extracellular vesicles
FBS: fetal bovine serum
FFPE: formalin‐fixed paraffin‐embedded
GEO: Gene Expression Omnibus
GST: glutathione S‐transferase
HEK: human embryonic kidney
hpf: high‐power field
ICBT: immune checkpoint blockade therapy
IFA: immunofluorescence assay
IHC: immunohistochemistry
IPA: ingenuity pathway analysis
IPTG: isopropyl Β‐D‐1‐thiogalactopyranoside
IRB: Institutional Review Board
MSI‐H: microsatellite instability‐high
MSS: microsatellite stable
NK: natural killer
PB: peripheral blood
PBS: phosphate‐buffered saline
PFA: paraformaldehyde
RT‐qPCR: real‐time quantitative PCR
RMST: restricted mean survival time
RNA‐seq: RNA‐sequencing
STR: short tandem repeat
TCGA: The Cancer Genome Atlas
Tfhs: T follicular helper cells
TME: tumor microenvironment
TPM: transcripts per million
Treg: regulatory T cell

## 1 Introduction

Colorectal cancer (CRC) has been classified into four consensus molecular subtypes (CMSs) based on different molecular and immune characteristics. Among these four subtypes, CMS1 is enriched in microsatellite instability‐high (MSI‐H) tumors bearing BRAF mutations. Tumors of the CMS1 subtype exhibit increased immune cell infiltration [1, 2]. MSI‐H tumors, accounting for up to 15% of all CRC cases, are characterized by defects in the DNA mismatch repair system (dMMR) and typically lose the expression of two of the four major proteins involved in the repair process: MLH1, MSH2, MSH6, and PMS2. Hypermethylation of the MLH1 gene promoter region is a leading cause of MMR protein deficiency and microsatellite instability [3]. Genome instability in dMMR tumors leads to an increased mutation burden and neoantigen formation, facilitating the recognition of tumor cells by cytotoxic lymphocytes. Therefore, MSI‐H status is a favorable prognostic marker and predicts a better treatment outcome [2]. Immune checkpoint blockade therapy (ICBT) can reactivate the immune system to attack and eliminate immunogenic MSI‐H tumors. MSI‐H CRC patients show a greater response rate to the immune checkpoint inhibitor anti‐PD1 antibody pembrolizumab than microsatellite stable (MSS) cancer patients, who mostly show a poor response [4]. The T‐cell‐mediated benefit of ICBT in metastatic MSI‐H CRC patient survival is superior to that of other treatment modalities [5]. However, in both studies, the tumor response rate to immunotherapy was only approximately 40%. Even when the anti‐PD1 antibody nivolumab was given with the anti‐CTLA4 antibody ipilimumab, the objective response rate was limited in MSI‐H CRC patients [6]. The efficacy of chemotherapy in stage III MSI‐H CRC patients is also variable [7], indicating that *de novo* or acquired resistance exists among MSI‐H tumors.

The dynamic tumor–host interaction in a spatially confined tumor microenvironment (TME) contributes to immune escape, one leading challenge for therapeutic heterogeneity [8, 9]. Tumor or host cell‐derived IL‐6 [10], TGF‐β [11], and extracellular vesicles (EVs) [12, 13], as well as glucose deprivation [14, 15] and lactate accumulation in the TME [16, 17], establish an immunosuppressive ‘cold’ niche to facilitate immune evasion. Although MSI‐H tumors have a ‘hot’ TME with significantly increased infiltration of B cells, CD8(+) T cells, activated memory CD4(+) T cells, T follicular helper cells (Tfhs), M1‐type macrophages, and neutrophils, while MSS tumors have greater regulatory T‐cell (Treg) infiltration [18, 19], decreased accumulation of CD56(+) natural killer (NK) cells in MSI‐H CRC tumors has been noted [20]. Biomarkers for determining cancer cell features and immune surveillance status in the TME of progressive MSI‐H CRC patients remain elusive.

In this study, we stratified MSI‐H CRC patients according to their survival outcomes and detected high CT45A1 expression in MSI‐H patients with poor survival outcomes. CT45A1‐expressing MSI‐H CRC cells, but not MSS CRC cells, exhibited enhanced resistance to NK cell cytotoxicity and generated homotypic cell‐in‐cell (CIC) structures with CT45A1‐expressing outer cells, protecting the inner cancer cells against therapeutic targeting. CT45A1 is a tumor intrinsic regulator that promotes aggressive behavior of MSI‐H CRC and poor clinical outcomes.

## 2 Materials and methods

### 2.1 Patient samples and clinical information

This study followed the guidelines of the Helsinki Declaration and was approved by the Institutional Review Board (IRB) of the Taipei Veterans General Hospital and Fu Jen Catholic University Hospital (VGHTPE 2020‐01‐010AC and FJUH111178). The experiments were undertaken with the understanding and written consent of each subject. A total of 272 CRC specimens were subjected to immunohistochemistry (IHC), survival analysis, and CIC structure quantification. 14 out of 272 CRC specimens were collected at Fu Jen Catholic University Hospital from September 2018 to October 2021, and the Biobank at the Taipei Veterans General Hospital provided the other specimens. The gene expression profiles and clinical information of the TCGA‐COAD and TCGA‐READ datasets deposited in The Cancer Genome Atlas (TCGA) were used to identify the differentially expressed genes among MSI‐H patients stratified based on the survival status.

### 2.2 Cell culture

DLD‐1 (RRID: CVCL_0248), HCT‐15 (RRID: CVCL_0292), and HT29 (RRID: CVCL_A8EZ) cells were cultured in RPMI 1640 medium (Gibco, Grand Island, NY, USA), and SW480 cells (RRID: CVCL_0546) were cultured in L15 medium (Gibco). Human embryonic kidney (HEK) 293 cells (RRID: CVCL_0045) were maintained in DMEM (Gibco). The above media were supplemented with 10% fetal bovine serum (FBS, Gibco) and 1% penicillin/streptomycin (Gibco). NK‐92MI cells (RRID: CVCL_3755) were cultured in alpha MEM (Gibco) supplemented with 12.5% FBS (HyClone, Logan, UT, USA), 12.5% horse serum (Gibco), 0.02 mm folic acid (Sigma‐Aldrich, St. Louis, MO, USA), 0.1 mm 2‐mercaptoethanol (Gibco), and 0.2 mm inositol (Sigma‐Aldrich). The above cells were initially purchased from ATCC. CD56(+)/CD16(+) human peripheral blood (PB) NK cells from one female donor (age 54) (2W‐501, Lonza Bioscience, Walkersville, MD, USA) were cultured in X‐VIVO™ 15 medium (Lonza Bioscience) supplemented with recombinant 10 ng·mL^−1^ IL‐2 (PeproTech, Cranbury, NJ, USA). The authenticity of the cell lines was verified by examining their DNA‐short tandem repeat (STR) profiles over the past 3 years, and all experiments were performed with mycoplasma‐free cells.

### 2.3 RNA extraction and real‐time quantitative PCR (RT‐qPCR)

The cell pellets were dissolved in TRIzol (Thermo Fisher, Waltham, MA, USA) for 20 min, and chloroform (Honeywell, Pittsburgh, PA, USA) was added for extraction. Isopropanol (Sigma‐Aldrich) was added to the collected aqueous phase to precipitate RNA on ice for 20 min, and the mixture was subsequently centrifuged at 12 000 ***g*** at 4 °C for 15 min. The washed RNA pellets were dissolved in nuclease‐free water (Invitrogen, Waltham, MA, USA). For complementary DNA (cDNA) preparation, template RNAs were mixed with dNTPs (Bioman, New Taipei City, Taiwan) and 1x random primers (Genestar, Kaohsiung City, Taiwan) at 65 °C for 5 min in a thermocycler (MultiGeneTM OptiMax Thermal Cycler, Labnet, Edison, NJ, USA). Then, 5× first‐strand buffer (Bionovas, Toronto, ON, Canada), 0.1 m DTT (Bionovas), and reverse transcriptase (Bionovas) were added and incubated at 30 °C for 10 min, 42 °C for 1 h, and 70 °C for 15 min. The diluted cDNA was mixed with SYBR Green Master Mix (Thermo Fisher) and the indicated primer sets. PCR was carried out with a StepOnePlusTM Real‐Time PCR System (Applied Biosystems Inc., Norwalk, CT, USA). The sequences of the primers used for RT‐qPCR are listed in Table S1.

### 2.4 RNA‐sequencing (RNA‐seq) and bioinformatics analysis

To retrieve TCGA datasets, we used the TCGAbiolinks package of the R program to query, download, and analyze the TCGA‐COAD and TCGA‐READ data [21]. Sixty MSI‐H CRC patients from the TCGA dataset were separated into favorable survival (*n* = 51 living patients) and unfavorable survival (*n* = 9 dead patients) groups based on their survival status to identify aggressive MSI‐H signatures under the following criteria: false discovery rate (FDR) < 0.01 and log2‐fold change ≥ 1 (accessed on May 2018). The functional clustering of enriched biological and molecular features was performed using the Database for Annotation, Visualization and Integrated Discovery (DAVID) (accessed on November 2023) [22]. For profiling CT45A1‐regulated transcriptome in CRC cells, a SureSelect XT HS2 mRNA Library Preparation Kit (Agilent, Santa Clara, CA, USA) was used for library construction, and the libraries were sequenced on a NovaSeq 6000 (Illumina, San Diego, CA, USA). The bcl2fastq conversion software v2.20 was used to obtain the FASTQ reads. Adaptor clipping and sequence quality trimming were performed using Trimmomatic v0.36, and HISAT2 was utilized for alignment. The transcript per million (TPM) values were generated using StringTie (StringTir v2.1.4). The RNA‐seq data of CT45A1‐expressing and vector control CRC cells were deposited in the Gene Expression Omnibus (GEO) under the accession number GSE193554. Ingenuity pathway analysis (IPA) was used to perform Gene Ontology (GO) analyses and to identify enriched signaling pathways (accessed on April 2023).

### 2.5 Immunoblotting and small GTPase pull‐down assay

The total protein concentration obtained from cultured cells was quantified with a BCA protein assay kit (Thermo Fisher) according to the manufacturer's instructions, and the lysates were then subjected to electrophoresis or pull‐down assays. To express GTPase effector proteins, *E. coli* competent cells were treated with 500 μm isopropyl β‐D‐1‐thiogalactopyranoside (IPTG, Sigma‐Aldrich) for 2 h to express GST‐tagged PAK1 and TRBD fusion proteins. The bacterial pellets were resuspended in 1 mL of 1% Triton X‐100 (Sigma‐Aldrich) and sonicated for 2 min under 40 amplitude with 20 s of pulse‐on and 20 s of pulse‐off using a Qsonica Q700 sonicator. GST‐tagged PAK1 (for RAC1‐GTP pull‐down) or GST‐tagged TRBD (for RHO‐GTP pull‐down) was incubated with 100 μL of glutathione (GSH) Sepharose (Cytiva, Marlborough, MA, USA) under a rotator at 4 °C for 1 h. Then, 250 μg of total cell lysate was added to GST‐protein bound beads at 4 °C for 1 h. The cleaned beads were subjected to western blotting as described previously [23] with the indicated antibodies. The immunoblots were visualized with an ImageQuant LAS 4000 chemiluminescence detection system (GE Healthcare Bio‐Sciences, Piscataway, NJ, USA) and quantified with imagej software. The antibodies used are listed in Table S2.

### 2.6 Flow cytometry analysis and immunofluorescence assay (IFA)

A total of 2 × 10^5^ cells were suspended in 100 μL of FACS buffer containing 1% bovine serum albumin (BSA, Bioshop, Burlington, ON, Canada) and 2 mm ethylenediaminetetraacetic acid (EDTA, J.T. Baker, Phillipsburg, NJ, USA) prepared in 1x phosphate‐buffered saline (PBS, Bioman) for hybridization with the following primary antibodies: APC‐conjugated anti‐CD107a (1 : 100, BioLegend, San Diego, CA, USA), Alexa Fluor 647‐conjugated anti‐HLA‐A, anti‐HLA‐B, anti‐HLA‐C (1 : 40, BioLegend), or APC‐conjugated anti‐PD‐L1 (1 : 40, BioLegend), on ice for 30 min. For Siglec‐7 ligand and Siglec‐9 ligand analysis, cells were incubated with recombinant Siglec7‐Fc protein (1 : 40, R&D Systems, Minneapolis, MN, USA) or recombinant Siglec9‐Fc protein (1 : 40, R&D Systems) on ice for 30 min and then hybridized with Alexa Fluor 647‐conjugated anti‐human IgG Fc antibodies (1 : 40, BioLegend) for 30 min. The cells were then washed with FACS buffer and fixed with 4% paraformaldehyde (PFA, Sigma‐Aldrich) at 4 °C for 30 min before analysis with a Beckman Coulter CytoFLEX flow cytometer. For the NK degranulation assay (CD107a analysis), 4 × 10^5^ NK‐92MI cells were cocultured with 4 × 10^5^ CRC cells preseeded in 6‐well plates overnight for 90 min before flow cytometry analysis. For the immunofluorescence assay (IFA), a total of 1.2 × 10^5^ CRC cells (6 × 10^4^ vector‐ and CT45A1‐expressing cells) were seeded into the wells of a 4‐well chamber slide (Thermo Fisher) in basal RPMI medium for 48 h, fixed with 4% PFA at room temperature (RT) for 20 min, and stained with APC‐conjugated anti‐PD‐L1 antibody (1 : 100, BioLegend), Alexa 647‐conjugated anti‐EGFR antibody (1 : 100, BioLegend), Alexa 647‐conjugated anti‐EPCAM antibody (1 : 100, ABclonal, Woburn, MA, USA), or unconjugated anti‐CD47 antibody (1 : 100, ABclonal) prepared in antibody dilution buffer (Ventana, Tucson, AZ, USA) on ice for 2 h. An Alexa 647‐conjugated secondary antibody (1 : 100, Cell Signaling Technology, Danvers, MA, USA) was used to recognize the unconjugated anti‐CD47 antibody on ice for 2 h. To examine membranous CD47 expression in the inner cells of CIC structures or the expression of CT45A1 and phosphorylated MLC2 (S19) in CRC cells, fixed cells were permeabilized with 0.1% Triton X‐100 at room temperature for 15 min and blocked in 5% BSA at room temperature for 60 min before antibody hybridization on ice for 2 h. The Alexa 647‐conjugated (1 : 100, Cell Signaling Technology) or Spark Red 718‐conjugated (1 : 100, BioLegend) secondary antibodies were then hybridized on ice for 2 h. The cells were then mounted with Fluoroshield containing DAPI (Sigma‐Aldrich) for imaging via confocal microscopy (LSM880, Zeiss, Oberkochen, Germany) and quantified with zen 2 software (blue edition, Zeiss). The antibodies used are listed in Table S2.

### 2.7 Lentivirus production and transduction

For virus production, 2 × 10^6^ HEK293 cells were seeded in 10 cm^2^ dishes and cultured overnight. A total of 2.5 μg of VSV‐G (National RNAi Core Facility, Academia Sinica, Taipei, Taiwan), 9 μg of ∆8.9 (National RNAi Core Facility, Academia Sinica), 10 μg of pLenti‐CT45A1 (OriGene, Rockville, MD, USA), pLenti‐Vector (OriGene) or MYLK shRNA plasmid (TRCN0000000937) and 20 μL of T‐pro NTR III transfection reagent (T‐Pro Biotechnology, New Taipei City, Taiwan) were mixed in 1 mL of basal DMEM for 30 min at room temperature and added dropwise to the cells. To generate mCherry and Venus‐expressing cells, 10 μg of pMDLg/pRRE (Addgene, Watertown, MA, USA), 5 μg of pRSV‐Rev (Addgene), 2 μg of VSV‐G, 10 μg of LeGO‐V2 (Venus, Addgene), or 10 μg of LeGO‐C2 (mCherry, Addgene) were transfected into HEK293 cells. The lentiviral particle supernatant was collected and transduced into the indicated cells with 8 μg·mL^−1^ polybrene (Sigma‐Aldrich) before cell sorting with a FACS Melody flow cytometer (BD Biosciences, Franklin Lakes, NJ, USA).

### 2.8 Cell viability and NK cell cytotoxicity assays

CRC cells (5 × 10^3^ per well) were seeded in 96‐well plates in complete RPMI medium for 72 h to measure cell growth. The medium was discarded, and a medium containing 5 mg·mL^−1^ thiazolyl blue tetrazolium bromide (MTT) reagent (Sigma‐Aldrich) was added to the cells for 45 min. MTT crystals were then dissolved in 100 μL of dimethyl sulfoxide (DMSO, Scharlau, Sentmenat, Barcelona, Spain), and the absorbance at 560 and 670 nm was measured with a microplate reader (Infinite M200 Pro, Tecan, Männedorf, Switzerland). For NK cell cytotoxicity analysis, 1 × 10^4^ CRC cells per well were seeded overnight in 96‐well plates and cocultured with NK‐92MI cells or human peripheral blood (PB) NK cells at different effector (E)/target (T) cell ratios for 4 h (DLD‐1, HCT‐15, and SW480) or 24 h (HT29) before the MTT assay. Y‐27632 (Cell Signaling Technology) and ML‐7 (Cayman Chemical, Ann Arbor, MI, USA) inhibitors were washed away from pretreated CRC cells before the NK cytotoxic assay. For studying the effect of CRC‐conditioned medium (CM) on NK activity, 2 × 10^6^ CRC cells were seeded in a 10 cm^2^ dish under complete medium cultivation for 72 h. The supernatant was harvested as conditioned medium, and 1.5 mL of CM was mixed with an equal volume of fresh NK‐92MI medium for treating 4 × 10^5^ NK‐92MI cells per well seeded in a 6‐well plate for 48 h before the cytotoxic assay.

### 2.9 Colony formation and Transwell migration assays

CRC cells (5 × 10^3^ per well) were seeded in 6‐well plates and cultured in complete RPMI medium for 7 days. Colonies were fixed with 4% PFA for 5 min and stained with 0.5% crystal violet (Sigma‐Aldrich) for 5 min. The number of colonies was quantified with imagej v1.52 software. To evaluate cell migration, 2 × 10^5^ CRC cells were suspended in 100 μL of basal medium and seeded in the upper chamber of a Transwell insert with 8 μm pores (Costar, Darmstadt, Germany). The lower wells were filled with 600 μL of RPMI medium supplemented with 20% FBS for chemoattraction. Twenty‐four hours after seeding, the cells were fixed with 4% PFA for 15 min and stained with 0.5% crystal violet for 15 min. Images were taken with a Nikon Eclipse TS2 microscope (Nikon Corporation, Tokyo, Japan) at 100× magnification to count the migrated cells.

### 2.10 CIC structures and time‐lapse tracking

To monitor homotypic CIC structure formation, 6 × 10^4^ CRC cells per well carrying Venus (V2) or mCherry (C2) proteins were suspended in basal medium and seeded in a 24‐well plate coated with 5 mg·mL^−1^ poly (2‐hydroxyethyl methacrylate) (Sigma‐Aldrich) with or without 100 μg·mL^−1^ IgG control antibody (R&D Systems) and anti‐PD‐L1 antibody (BioXcell, Lebanon, NH, USA) for 48 h for quantifying the CIC structures using an Olympus IX83 inverted microscope (Olympus Corporation, Tokyo, Japan) equipped with a humidified cell chamber with 5% CO_2_. An established homotypic CIC structure was defined as more than 50% of an inner cell enclosed within an outer cell under 200× magnification. A total of 6 × 10^4^ CFSE‐stained SW480 CT45A1‐expressing cells were cocultured with 6 × 10^4^ unlabeled vector‐expressing cells in basal DMEM for 72 h and fixed to quantify the CIC structures with FV1000 confocal microscopy (Olympus). To visualize the heterotypic CIC structure, 1.2 × 10^5^ unlabeled NK‐92MI cells were added to 6 × 10^4^ preseeded CRC cells (mCherry carrying vector cells or Venus carrying CT45A1‐expressing CRC cells) per well on wells of a 24‐well plate for cell tracking at 5‐min image intervals using an Olympus IX83 inverted microscope. The numbers of CIC structures counted are shown in the corresponding figure legends. The killing of CRC cells was defined by the loss of a fluorescent signal. To quantify NK cell cytotoxicity, a total of 1.2 × 10^5^ CRC cells (6 × 10^4^ mCherry‐expressing vector‐ and Venus‐expressing CT45A1‐expressing cells) were cultured overnight in a 24‐well plate, and 1.2 × 10^5^ NK‐92MI cells were added per well for cell tracking with 30‐min image intervals using an Olympus IX83 inverted microscope.

### 2.11 Immunohistochemistry (IHC) and quantification of CIC structures

The deparaffinized tissue sections were autoclaved in 10 mm citric acid (Honeywell) buffer for antigen retrieval at pH 6.0. The sections were then immersed in 3% H_2_O_2_ for 10 min, followed by 0.1% Triton X‐100 for 5 min at room temperature. Primary antibodies against CT45A1 (1 : 200, Novus Biologicals, Centennial, CO, USA) and phospho‐MLC‐2 (S19) (1 : 50, Cell Signaling Technology) were diluted with antibody dilution buffer (Ventana) and hybridized at 4 °C overnight. The tissue sections were washed three times and incubated with anti‐immunoglobulin cocktails (BioGenex, Fremont, CA, USA) for 30 min at room temperature and then with streptavidin peroxidase (BioGenex) for 20 min at room temperature. DAB solution (Epredia, Kalamazoo, MI, USA) was used for visualization. Sections were counterstained with Mayer's hemalum solution (Sigma‐Aldrich) and mounted with Kaiser's glycerol gelatin mounting medium (Millipore, Burlington, MA, USA). The histology score (H‐score) was defined as the percentage of the CT45A1‐positive immunostained region (0 to 100) multiplied by the intensity of CT45A1 staining (0, 1, 2, and 3). An H‐score greater than 150 indicated positive immunoreactivity. Hematoxylin and eosin (HE)‐stained sections were examined under 400x magnification to quantify CIC structures in 10 high‐power fields (hpf). Structures within this examined area that fulfilled at least four of the six criteria were considered CIC structures, and sections with at least one CIC structure per 10 hpf were considered positive. The six criteria for counting CICs were as follows: (a) The nucleus of the internalized cell was visible. (b) The cytoplasm of the internalized cell was visible. (c) The nucleus of the engulfing cell was visible. (d) The cytoplasm of the engulfing cell was visible. (e) The nucleus of the engulfing cell showed a moon‐shaped deformity. (f) A vacuolar space is identified between the internalized cell and the engulfing cell. Images were evaluated with an Olympus BX43 microscope equipped with a DP22 CCD camera (Olympus). All reagents and chemicals used are summarized in Table S3.

### 2.12 Statistical analysis

spss software (version 16.0; IBM, Armonk, NY, USA) or graphpad prism (version 9.5.1; Dotmatics, Boston, MA, USA) was used for the statistical analyses. Single biological replicates or single‐cell analyses are shown as dots. The normality of data was checked by the Shapiro–Wilk test, and two‐sided Student's *t*‐test or ANOVA followed by Tukey's *post hoc* test was used to assess the significance. The Mann–Whitney *U* test was performed when the data did not follow a normal distribution. Fisher's exact test was used to analyze correlations between clinicopathological variables and CT45A1 immunopositivity. Survival was estimated using Kaplan–Meier plots with the log‐rank test or restricted mean survival time (RMST) analysis (r Package: survrm2, https://www.R-project.org/). The data are presented as the means ± SDs or SEMs, as indicated in the corresponding figure legends. *P* values less than 0.05 were considered significant.

## 3 Results

### 3.1 Identification of the aggressive MSI‐H CRC signature in TCGA datasets

**Fig. 1:**
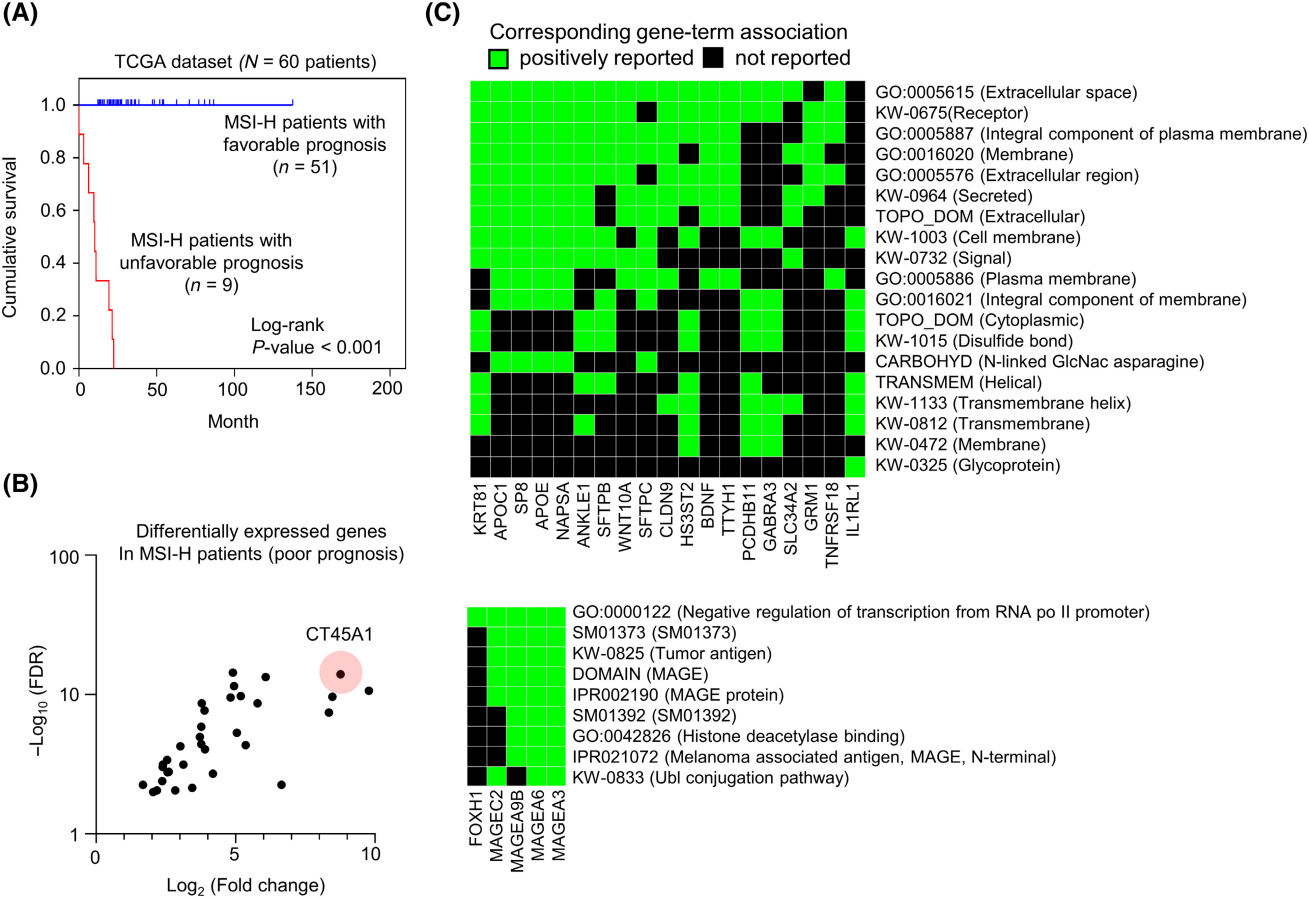
Identification of CT45A1 in aggressive MSI‐H CRC patients. (A) Kaplan–Meier plot depicting the overall survival of 60 MSI‐H CRC patients with poor and favorable prognoses in the TCGA dataset. The *P* value was estimated by the log‐rank test. (B) Volcano plot illustrating the differentially expressed genes in MSI‐H patients with a poor prognosis. Black dots, the differentially expressed genes. FDR, false discovery rate. Red shading, the *CT45A1* gene. (C) Heatmaps showing the enriched subcellular location (upper panel) and biological function (lower panel) of the aggressive MSI‐H CRC signature.

In an attempt to understand the mechanisms governing the variability in MSI‐H CRC patient outcomes, we stratified 60 MSI‐H CRC patients annotated in the TCGA dataset based on their vital status into favorable (alive) and unfavorable (dead) survival subsets (Fig. 1A) and a total of 33 upregulated genes in unfavorable MSI‐H CRC patients (FDR < 0.01 and log2‐fold change ≥ 1) were identified as the aggressive MSI‐H CRC signature (Fig. 1B and Table S4). Genes involving the extracellular space and plasma membrane regulators were increased in aggressive MSI‐H CRC tumors (Fig. 1C, upper panel), and tumor antigens (MAGEA3, MAGEA6, MAGEA9B, or MAGEC2) involving in the transcriptional and post‐translational regulations were represented in the aggressive MSI‐H signature (Fig. 1C, lower panel). However, CT45A1, a highly expressed tumor antigen with a relatively low FDR among the other differentially expressed (DE) genes in the aggressive MSI‐H CRC tumors (Fig. 1B and Table S4), was not annotated in the functional enrichment analysis (Fig. 1C). Therefore, we aimed to elucidate the biological and clinical relevance of CT45A1 during advanced MSI‐H CRC progression.

### 3.2 Ectopic CT45A1 expression in MSI‐H CRC cells promotes resistance to NK cell cytotoxicity

**Fig. 2:**
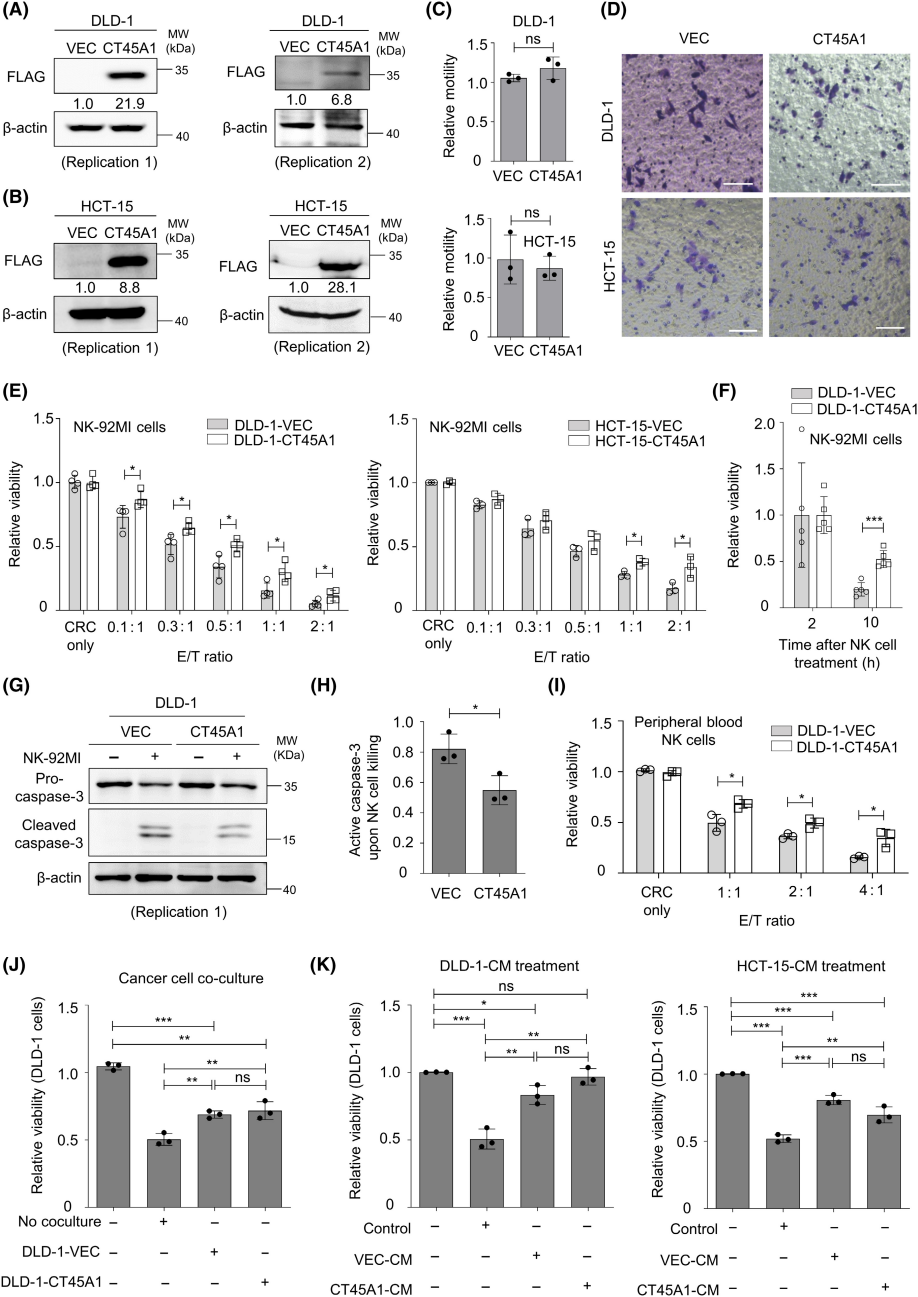
Overexpression of CT45A1 in MSI‐H CRC cells promotes resistance to NK cell cytotoxicity. (A, B) Western blot images showing the expression of myc‐DDK‐tagged CT45A1 in DLD‐1 (A) and HCT‐15 (B) cells using an anti‐FLAG antibody. MW, molecular weight. Two biological replicates are shown. (C) Histograms showing the relative motility of CRC cells in a Transwell assay. *n* = 3. (D) Representative images showing the migrated cells in (C). Scale bar = 100 μm. (E) MTT analysis showing the sensitivity of cancer cells to NK‐92MI cells at the indicated effector (E)/target (T) cell ratios. *n* = 4 for DLD‐1 cells. *n* = 3 for HCT‐15 cells. (F) Histogram showing the sensitivity of DLD‐1 cells to NK‐92MI cells examined by an IX83 time‐lapse fluorescence microscope. h, hour. *n* = 5. (G) Immunoblots showing the expression of pro‐caspase‐3 and cleaved caspase‐3 with or without coculture with NK‐92MI cells for 90 min. MW, molecular weight. Representative images from three experiments (*n* = 3). (H) Quantification of cleaved caspase‐3 upon NK cell killing. *n* = 3. (I) MTT analysis showing the sensitivity of target cancer cells to human NK cells at the indicated effector (E)/target (T) cell ratios. *n* = 3. (J) MTT analysis showing the viability of DLD‐1 cells (the second‐round killing) after culture with the NK‐92MI cells cocultured with indicated CRC cells during the first‐round killing assay. *n* = 3. (K) MTT analysis showing the viability of DLD‐1 cells treated with the indicated cancer‐conditioned medium (CM)‐educated NK‐92MI cells. *n* = 3. The data are presented as the means ± SDs, and *P* values were determined by Student's *t*‐test for (C), (E), (F), (H), (I), (J), and (K). *P < 0.05; **P < 0.01; and ***P < 0.001. ns, nonsignificant.

Because CT45A1 is upregulated in aggressive MSI‐H CRC tumors, we next ectopically expressed CT45A1 in MSI‐H CRC cell lines for functional characterization. The expression of CT45A1 in DLD‐1 (Fig. 2A) and HCT‐15 (Fig. 2B) cells was confirmed. We found that the expression of CT45A1 in these two MSI‐H CRC cell lines did not consistently affect cell viability (Fig. S1A), clonogenicity (Fig. S1B), epithelial–mesenchymal transition (EMT)‐related gene expression (Fig. S1C), Transwell motility (Fig. 2C,D), or stemness gene expression (Fig. S1D). Because aggressive MSI‐H CRC may result from escaping the immune surveillance of cytotoxic lymphocytes and NK cell is a type of cytotoxic immune cells, which target cancer cells in an antigen‐independent manner [24], we use NK‐92MI cells as a model cell line to explore the roles of CT45A1 in the cancer susceptibility to cytotoxic killing. Both MSI‐H CT45A1‐overexpressing DLD‐1 and HCT‐15 cells showed higher resistance to NK‐92MI cells compared to vector‐transduced cells (Fig. 2E). According to the time‐lapse imaging, when Venus‐labeled CT45A1‐expressing DLD‐1 cells were cocultured with mCherry‐labeled vector cells in the presence of NK‐92MI cells, more CT45A1‐expressing cells survived at the end of tracking (Fig. 2F). Reduced levels of cleaved (active) caspase‐3 in CT45A1‐expressing CRC cells cultured with NK‐92MI cells were noted (Fig. 2G,H and Fig. S1E). Consistently, enhanced resistance of CT45A1‐expressing CRC cells to human CD56(+)/CD16(+) PB‐NK cells was observed (Fig. 2I).

### 3.3 Ectopic CT45A1 expression in MSI‐H CRC cells does not modulate the cytotoxic activity of NK cells

The increased resistance to NK killing in CT45A1‐overexpressing CRC cells may be a result of intrinsic resistance of the cancer cells themselves or decreased cytotoxic activity of the cocultured NK cells. The balance of NK activation, inhibitory signaling, and cancer sialylation regulates the vulnerability of target cancer cells to NK cell killing [25, 26]. We then examined the inhibitory ligands that drive NK cell suppression in CRC cells. Ectopic CT45A1 expression in two MSI‐H CRC cell lines did not alter the surface expression of MHC‐I (Fig. S2A,B), PD‐L1 (Fig. S2C,D), or sialylated ligands for Siglec‐7 (Fig. S2E,F) or Siglec‐9 (Fig. S2G,H). Enhanced CD107a expression (degranulation) in NK‐92MI cocultured with cancer cells (first‐round cytotoxic assay) was observed compared to that in naïve NK cells (Fig. S3A–C), and these degranulated NK‐92MI cells showed impaired killing activity in the second‐round cytotoxic assay (Fig. 2J). However, compared with those of vec CRC‐coculturing NK‐92MI cells, the degranulation of NK‐92MI cells cocultured with CT45A1‐expressing DLD‐1 cells (Fig. S3B) and HCT‐15 cells (Fig. S3C) and the second‐round cytotoxicity of cocultured NK‐92MI cells were not further suppressed (Fig. 2J). Similarly, NK‐92MI cells treated in conditioned medium (CM) from both vec‐ and CT45A1‐expressing CRC cells for 48 h showed reduced cytotoxicity compared to control NK cells (Fig. 2K), but CM from CT45A1‐expressing CRC cells did not further suppress NK cell cytotoxicity compared to that from vector control cells (Fig. 2K). Overall, the survival advantage of CT45A1‐expressing MSI‐H CRC cells during NK cell killing may not be a result of decreased activity of NK cells, highlighting that CT45A1 is a cancer‐autologous driver of NK cell resistance.

### 3.4 CT45A1 expression in MSI‐H CRC cells resists the in‐cell NK killing and generates a protective homotypic CIC structure, with CT45A1‐expressing cancer cells being the outer cell

**Fig. 3:**
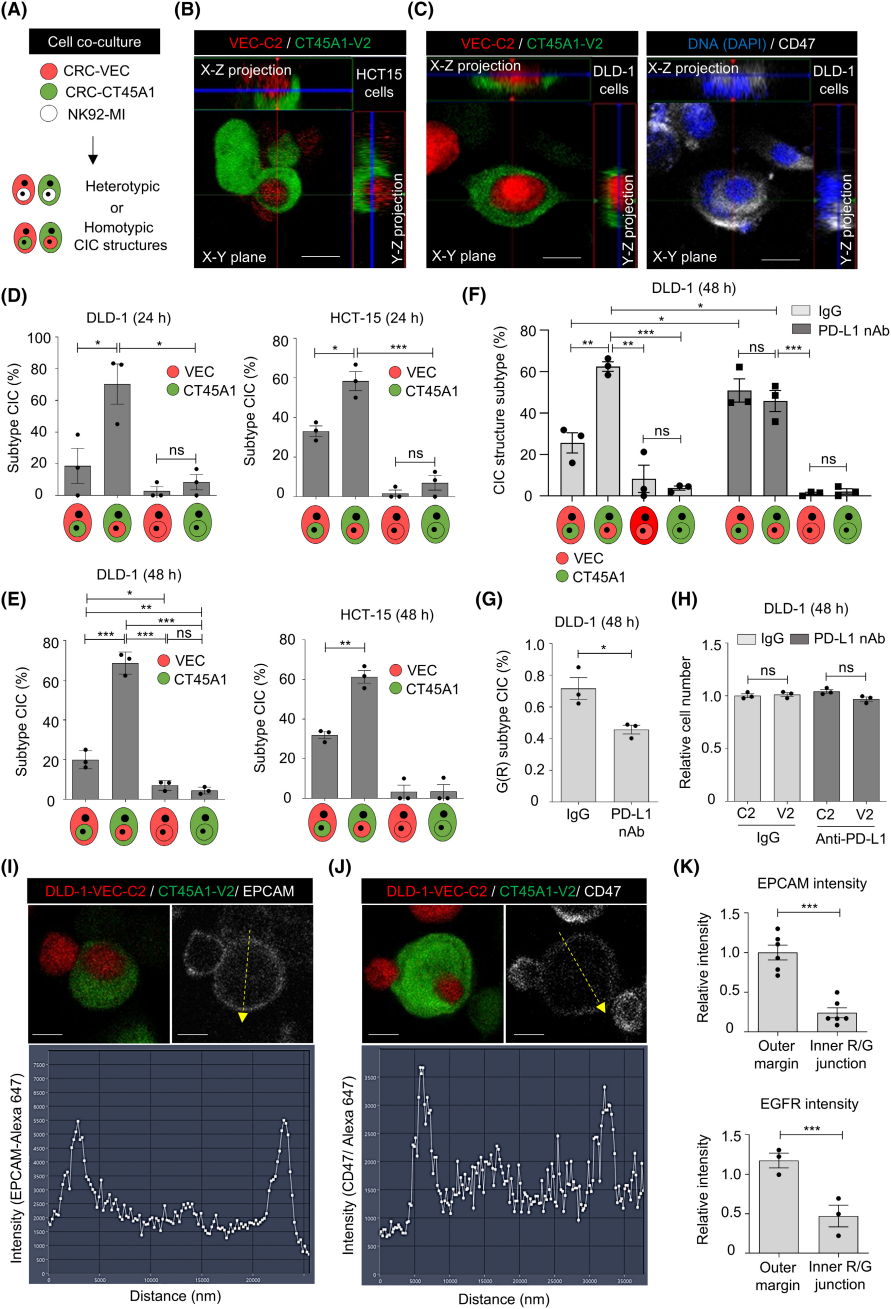
CT45A1 overexpression engenders an outer cell fate in a homotypic cell‐in‐cell (CIC) structure. (A) Schematic illustrating the experimental flow chart for examining heterotypic and homotypic CIC structures. Red, vec‐mCherry (C2)‐expressing cancer cells; green; CT45A1‐Venus (V2)‐expressing cancer cells; and white, unlabeled NK‐92MI cells. (B, C) Confocal images showing X‐Y and Y‐Z projection images of a CIC structure. Scale bar = 10 μm. (D–E) Subtypes of CIC structures observed at 24 h (D) and 48 h (E). h, hours. There were 29 CIC structures in DLD‐1 cells at 24 h, 41 in HCT‐15 cells at 24 h, 83 in DLD‐1 cells at 48 h, and 68 in HCT‐15 cells at 48 h. *n* = 3. (F) Subtypes of CIC structures in the presence of antibody (100 μg·mL^−1^) upon cell seeding for 48 h. The number of CIC structures was 85 (IgG control) and 79 (anti‐PD‐L1 group). *n* = 3. (G) Histograms showing the CIC structure frequency of the G(R) CIC structure subtype (outer green CT45A1‐expressing cells with inner red vec‐expressing cells) after antibody treatment. nAb, neutralizing antibody. *n* = 3. (H) Histograms showing cell viability upon anti‐PD‐L1 treatment. V2, Venus‐carrying, CT45A1‐expressing cells and C2, mCherry‐carrying, vec‐expressing cells. *n* = 3. (I, J) Upper: Representative images showing the labeling of established CIC structures with anti‐EPCAM (I) and anti‐CD47 (J) antibodies. The dashed‐line arrow indicates the intensity path across a CIC structure in which the fluorescence signal is quantified (arrow, end of the path direction). Scale bar = 10 μm. Lower: Plots showing fluorescence intensity along the dashed‐line (from the arrow tail to the arrowhead) in the corresponding upper right panel. Representative images from three experiments (*n* = 3). (K) Relative fluorescence intensity of CIC structures. The outer margin is the average peak intensity at both ends in the quantification path; the inner R/G junction is the fluorescence intensity at the cell contact interface in a CIC structure. *n* = 6 CIC structure (upper panel) and 3 (lower panel). The data are presented as the means ± SEMs, and *P* values were determined by Student's *t*‐test for (D), (E), (F), (G), (H), and (K). *P < 0.05; **P < 0.01; and ***P < 0.001. ns, nonsignificant.

CIC describes a phenomenon where one cell is located within another, forming unique structures reminiscent of bird's eyes [27]. The homotypic CIC structure, which usually involves two cancer cells, has been found to drive cell aneuploidy and foster cellular competition, thereby influencing tumor advancement [27]. Heterotypic CIC structures specifically refer to incorporating one type of living cell into another cell type [27, 28]. As the heterotypic CIC structure generated from the engulfment of NK cells by cancer cells potentiates the in‐cell NK killing [28], we explored the presence of encapsulated NK cells in CRC cells during NK cell killing (Fig. 3A). Heterotypic CIC structures generated by unlabeled NK‐92MI cells and mCherry‐labeled, vec‐expressing CRC cells or Venus‐labeled, CT45A1‐expressing cells were observed 1 h after NK cell seeding (Fig. S4A, white arrows), and unexpectedly, enhanced heterotypic CIC structures were observed when NK‐92MI cells were cultured with CT45A1‐expressing CRC cells (Fig. S4B). Time‐lapse tracking revealed that more CT45A1‐expressing CRC cells with internalized NK cells were alive (Fig. S4C), although most CRC cells were dead upon NK cell contact without the appearance of heterotypic CIC structures (defined as kiss killing) (Fig. S4D). Moreover, the homotypic CIC structure of two cancer cells could be observed upon coculturing Venus‐carrying, CT45A1‐expressing MSI‐H CRC cells with mCherry‐carrying, vector‐expressing MSI‐H CRC cells (Fig. S5A). Internalized inner cancer cells and crescent‐shaped outer cancer cells were confirmed using confocal microscopy (Fig. 3B). Double layers of CD47(+) membranes were observed in a homotypic CIC structure (Fig. 3C). The frequency of homotypic CIC structure formation was approximately 0.5–1.5% from 24 to 48 h (Fig. S5B), and no consistent changes in cell viability were observed during cell cultivation (Fig. S5C,D). We found that the CT45A1‐expressing MSI‐H CRC cells tended to be the outer cells in CIC structures at 24 h (Fig. 3D) and 48 h (Fig. 3E) after cell seeding. Furthermore, the initial administration of anti‐PD‐L1 antibodies to PD‐L1‐overexpressing MSI‐H DLD‐1 cells (Fig. S2C,D) upon cell seeding suppressed the frequency of the homotypic CIC structure subtype with CT45A1‐expressing cells being outer cells (Fig. 3F,G and Fig. S5E) without altering cell viability (Fig. 3H), suggesting the requirement of physical contact, which can be interfered by cell surface decoration by antibodies like anti‐PD‐L1, for this kind of CIC structure formation at least in part. The administration of anti‐PD‐L1 antibodies did not promote CT45A1 expression in MSI‐H DLD‐1 cells (Fig. S5F). When probing surface proteins with specific antibodies in established CIC structures without cell permeabilization, we found that regions of the engulfed inner vector‐expressing MSI‐H CRC cells were not labeled with anti‐EPCAM antibody (Fig. 3I and Fig. S6A, left), anti‐EGFR antibody (Fig. S6B), anti‐CD47 antibody (Fig. 3J and Fig. S6A, right), or anti‐PD‐L1 antibody (Fig. S6C). The fluorescence intensities of anti‐EPCAM (Fig. 3K, upper) and anti‐EGFR (Fig. 3K, lower) antibodies were lower at the cell contact point in homotypic CIC structures than on the outer cell surface, suggesting that NK cell‐resistant CT45A1‐expressing outer MSI‐H CRC cells may shield inner cancer cells from the recognition of therapeutic antibodies, contributing to spatial therapeutic resistance.

### 3.5 The activated RHO‐ROCK/MLCK‐MLC2 pathway mediates NK cell resistance and homotypic CIC structure formation in CT45A1‐expressing MSI‐H CRC cells

**Fig. 4:**
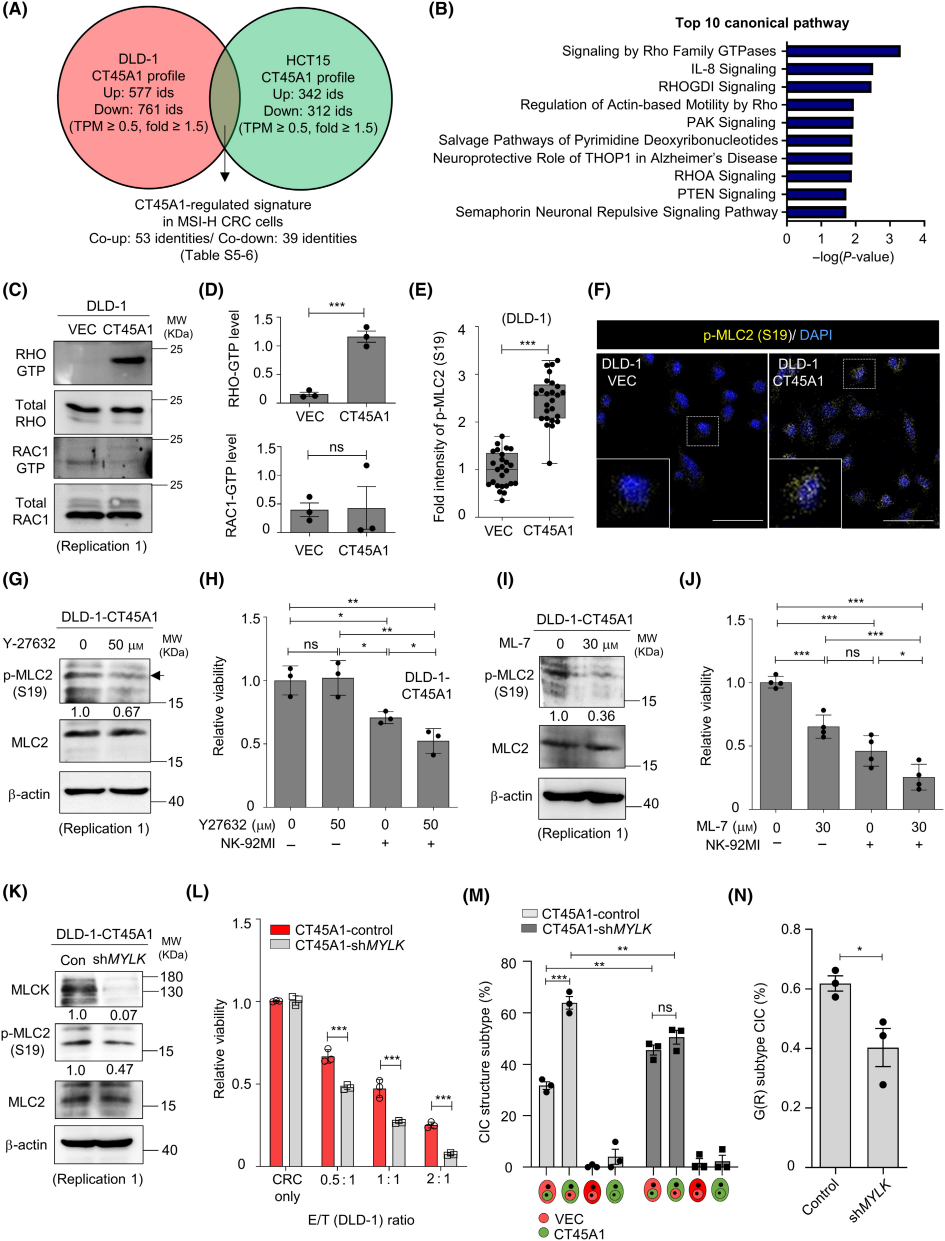
CT45A1 increases MLC2 phosphorylation in MSI‐H CRC cell lines. (A) Venn diagrams showing genes in two CRC cell lines regulated by CT45A1. Genes with at least 0.5 transcripts per million (TPM) in either control or CT45A1‐expressing cells and showing at least a 1.5‐fold change in TPM between two conditions were considered differentially expressed and included in the CT45A1 profile. (B) Gene Ontology analysis by IPA showing the top 10 molecular categories enriched in CT45A1‐expressing MSI‐H CRC cells. (C) Western blot images showing RHO‐GTP and RAC1‐GTP levels in DLD‐1 cells. MW, molecular weight. Representative images from three experiments (*n* = 3). (D) Quantification of RHO‐GTP and RAC1‐GTP levels in DLD‐1 cells. *n* = 3. (E) Fold change of phosphorylated MLC2 (S19) intensity determined by IFA. There were 25 single Vec‐expressing cells and 25 CT45A1‐expressing CRC cells. (F) Representative immunofluorescence confocal images of phosphorylated MCL2 (S19) expression. Scale bar = 20 μm. The lower left inserts are higher magnification images in corresponding dashed squares. Representative images from three experiments (*n* = 3). (G) Western blot images showing the level of phosphorylated MLC2 (S19) in CT45A1‐expressing cells treated with the ROCK inhibitor Y27632 for 24 h. Representative images from two experiments (*n* = 2). (H) Relative viability of CRC cells pretreated with Y‐27632 for 48 h before NK‐92MI cell killing. *n* = 3. (I) Western blot images showing MLC2 (S19) phosphorylation in cancer cells treated with the MLCK inhibitor ML‐7 for 24 h. Representative images from two experiments (*n* = 2). (J) Relative viability of CRC cells pretreated with ML‐7 for 24 h before NK‐92MI cell killing. *n* = 3. (K) Immunoblots showing MLCK expression and MLC2 phosphorylation upon *MYLK* silencing. Representative images from two experiments (*n* = 2). (L) Histogram showing the increased vulnerability of *MYLK*‐knockdown cancer cells to NK‐92MI cells in a background of CT45A1 overexpression. *n* = 3. (M) Histogram showing CIC structure subtypes in the indicated cells. The number of CIC structures was 109 for the control cells and 58 for the sh*MYLK* group. *n* = 3. (N) Histograms showing the CIC structure frequency of the G(R) CIC structure subtype. *n* = 3. The data are presented as the means ± SDs for (D), (E), (H), (J), and (L). The data are presented as the means ± SEMs for (M) and (N). *P* values were determined by Student's *t*‐test for (D), (E), (J), and (N). *P* values were determined by one‐way ANOVA followed by Tukey's *post hoc* test for (H) and two‐way ANOVA followed by Tukey's *post hoc* test for (L) and (M). *P < 0.05; **P < 0.01; and ***P < 0.001. ns, nonsignificant.

To explore the mechanism governing NK cell resistance and CIC structure formation in MSI‐H CRC cells, the differentially expressed CT45A1 signature was identified in CT45A1‐expressing DLD‐1 and HCT‐15 cell lines (Fig. 4A and Tables S5 and S6). RHO family GTPase and RHOGDI signaling, as well as actin‐based motility regulation by Rho, were the most enriched pathways in CT45A1‐expressing MSI‐H CRC cells (Fig. 4B). As RHO and RAC1 reciprocally regulate the actin cytoskeleton by modulating MLC2 phosphorylation [29] and CIC structure formation [27], we examined the RHO/RAC1 activity in CRC cells using a pull‐down assay. Increased RHO‐GTP but not RAC1‐GTP levels (Fig. 4C,D and Fig. S7A,B) and elevated MLC2 phosphorylation (Fig. 4E,F and Fig. S7C) were observed in DLD‐1‐CT45A1 cells. Inactivation of ROCK signaling with a Y‐27632 inhibitor abolished MLC2 phosphorylation (Fig. 4G and Fig. S7D, left) and increased the vulnerability of DLD‐1‐CT45A1 cells to NK‐92MI cytotoxicity (Fig. 4H), indicating the activation of RHO‐ROCK‐MLC2 signaling in CT45A1‐expressing MSI‐H CRC cells. MLC2 can also be phosphorylated by MLCK [29]. Treating DLD1‐CT451A1 cells with the MLCK inhibitor, ML‐7, decreased MLC2 phosphorylation (Fig. 4I and Fig. S7D, right) and promoted cancer cell vulnerability to NK cell killing (Fig. 4J). Treating HCT15‐CT45A1 cells with ML‐7 also decreased MLC2 phosphorylation (Fig. S7E) and sensitized cancer cells to NK cell killing (Fig. S7F), suggesting that MLCK activation also occurs in CT45A1‐expressing CRC cells. Moreover, knocking down *MYLK* (the gene encoding MLCK) expression with a specific shRNA reduced MLC2 phosphorylation (Fig. 4K and Fig. S7G) and sensitized DLD‐1‐CT451A1 cells to NK cell cytotoxicity (Fig. 4L). The percentage of homotypic CIC structure subtypes with CT45A1‐overexpressing cells as the outer cell was reduced when MLCK was silenced in the CT45A1‐overexpressing cells (Fig. 4M,N). Overall, MLC2 phosphorylation is required for NK cell resistance and outer cell fate in CIC structures of CT45A1‐overexpressing MSI‐H CRC cells.

### 3.6 CT45A1 overexpression promotes migratory capacity but not NK cell resistance in MSS CRC cells

**Fig. 5:**
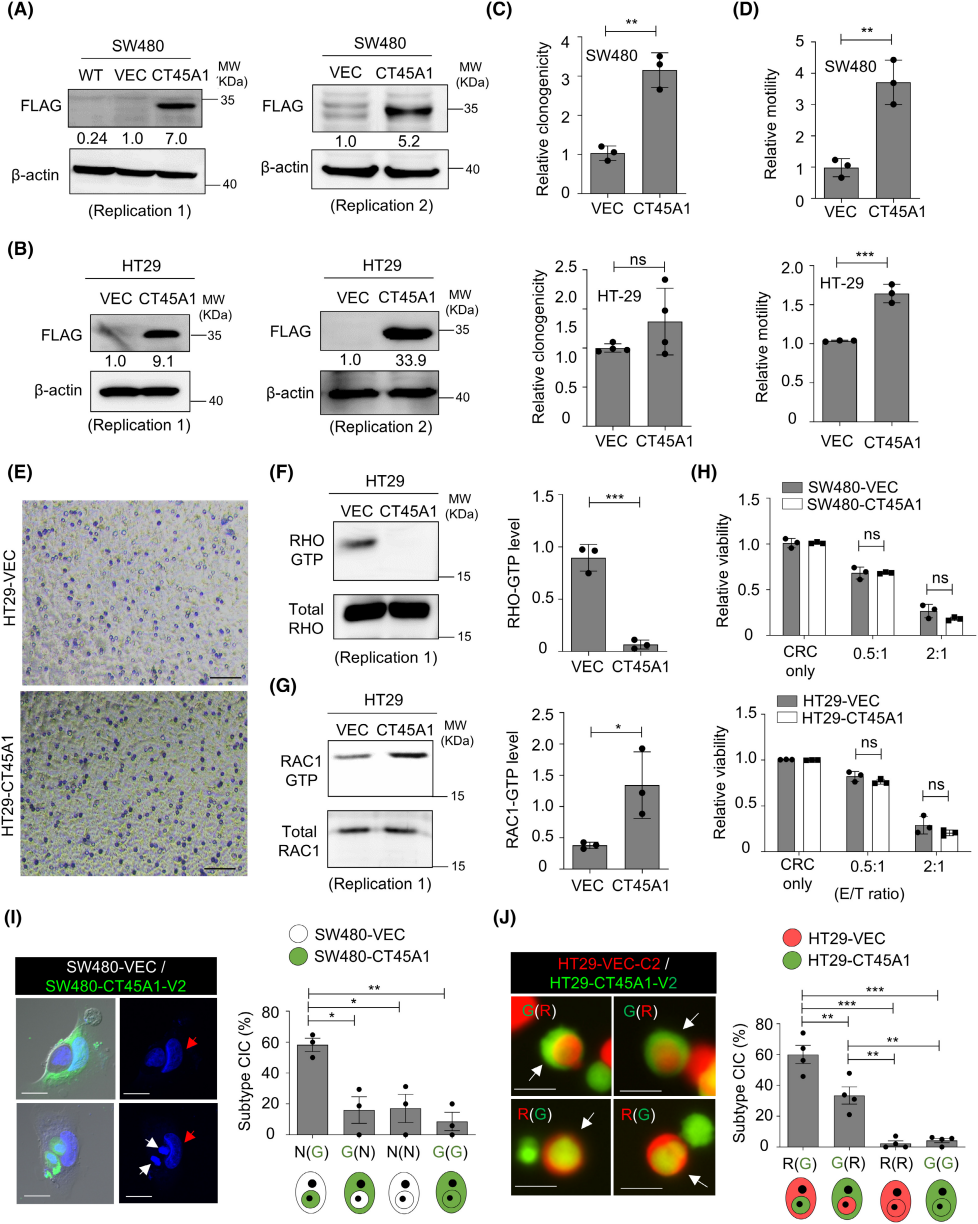
Increased migratory ability and inner cell fate in MSS CRC cells with ectopic CT45A1 expression. (A, B) Western blot analysis showing the expression of Myc‐DDK‐tagged CT45A1 in SW480 cells (A) and HT29 cells (B) with an anti‐FLAG antibody. MW, molecular weight. *n* = 2. (C, D) Histograms illustrating clonogenicity (C) and Transwell migration ability (D) of the cells. *n* = 3 (upper panel in C and both panels in D). *n* = 4 (lower panel in C). (E) Representative images of migrated cells in the Transwell assay. Scale bar = 50 μm. (F, G) Left: Immunoblots showing the RHO‐GTP (F) and RAC1‐GTP (G) levels among the groups. Right: Quantification results. *n* = 3. (H) The cancer cell viability in the presence of NK‐92MI cells. *n* = 3. (I) Left: Images showing CIC structures from SW480 cells. Red arrows, outer cancer cells and white arrows, inner cancer cells. Scale bar = 10 μm. Right: Percentage of CIC structure subtypes in SW480 cells at 72 h. CT45A1‐expressing SW480 cells were labeled with CFSE dye (green). N(G), CIC structures with outer unlabeled vector cells and inner CFSE‐labeled CT45A1‐expressing cells. *n* = 38 CIC structures were counted. *n* = 3. (J) Left: Images showing CIC structures from HT29 cells. White arrows, CIC structures. R, HT29‐vec‐c2 cells; G, HT29‐CT45A1‐V2 cells; R(G), CIC structures with outer vector red cells and inner CT45A1‐expressing green cells. Scale bar = 10 μm. Right: Percentage of CIC structure subtypes in HT29 cells at 48 h. *n* = 217 CIC structures were counted. *n* = 4. The data are presented as the means ± SDs for (C), (D), (F, right panel), (G, right panel), and (H). The data are presented as the means ± SEMs for (I, right panel) and (J, right panel). *P* values were determined by Student's *t*‐test for (C), (D), (F, right panel), (G, right panel), and (H). *P* values were determined by one‐way ANOVA followed by Tukey's *post hoc* test for (I) and (J). *P < 0.05; **P < 0.01; and ***P < 0.001. ns, nonsignificant.

To examine the effects of CT45A1 on MSS CRC cells, we first examined the endogenous level of CT45A1 in MSS CRC cells. As a low expression level of CT45A1 was detected in MSS CRC cell panels (Fig. S8A), we overexpressed CT45A1 in two CRC cell lines with MSS features (SW480 and HT29) (Fig. 5A,B). We found that clonogenicity was not consistently altered in CT45A1‐expressing MSS CRC cells (Fig. 5C). Enhanced migratory ability (Fig. 5D,E), reduced RHO‐GTP (Fig. 5F and Fig. S8B), and elevated RAC1‐GTP levels (Fig. 5G and Fig. S8C) were noted in HT29‐CT45A1 cells. Moreover, neither CT45A1‐expressing SW480 nor CT45A1‐expressing HT29 cells were more resistant to NK cell killing compared to vector‐expressing CRC cells (Fig. 5H), and the above cells exhibited an inner cell fate in homotypic CIC structures (Fig. 5I,J). Here, CT45A1 expression may give rise to different phenotypes of CRC cells with MSI‐H and MSS characteristics.

### 3.7 CT45A1 expression is closely associated with MLC2 phosphorylation, outer cell fate in a homotypic CIC structure, and unfavorable survival in MSI‐H CRC patients

**Fig. 6:**
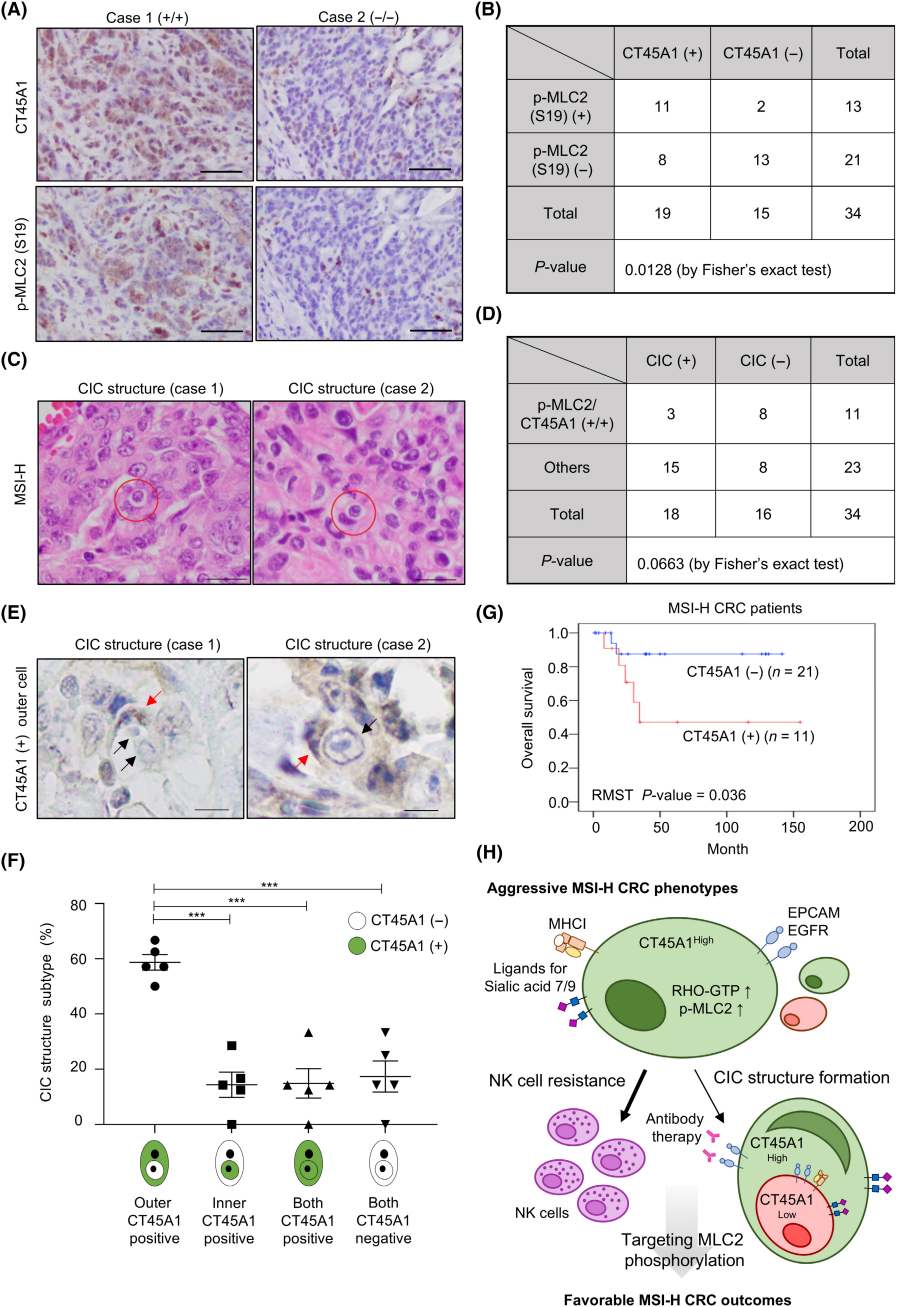
CT45A1 expression is positively correlated with MLC2 phosphorylation and marks the outer cells of homotypic CIC structures in MSI‐H CRC patients. (A) Representative images showing immunoreactivity for CT45A1 and MLC2 (S19) phosphorylation in MSI‐H CRC tumors. Scale bar = 50 μm. *n* = 2. (B) A table summarizing the correlation between the immunoreactivities of CT45A1 and MLC2 (S19) phosphorylation. Fisher's exact test was used to estimate the *P* value. (C) Images showing CIC structures by HE staining in MSI‐H CRC patients. Red circles, CIC structures identified. Scale bar = 20 μm. (D) A table summarizing the correlation of CIC structure positivity with the immunoreactivities of CT45A1 and MLC2 (S19) phosphorylation. Fisher's exact test was used to estimate the *P* value. (E) Representative images showing the expression of CT45A1 in the indicated CIC structures. Red arrows, outer cells with upregulated CT45A1 expression and black arrows, inner cells with low CT45A1 expression. Scale bar = 20 μm. (F) Histogram showing the distribution of CT45A1 expression in CIC structures. A total of 36 CIC structures were counted from five MSI‐H cases. Outer CT45A1 positive, CIC structures with upregulated CT45A1 expression in outer cells; inner CT45A1 positive, CIC structures with upregulated CT45A1 expression in inner cells; both CT45A1 positive, both outer and inner cells are CT45A1‐positive cells; and both CT45A1 negativity, both outer and inner cells are CT45A1‐negative cells. The data are presented as the means ± SEMs. *P* values were determined by one‐way ANOVA followed by Tukey's *post hoc* test. ***P < 0.001. (G) Kaplan–Meier plot depicting the overall survival of 32 MSI‐H CRC patients with survival information in the collected tissue banks. The *P* value was estimated by RMST. (H) Schematic representation of the mechanisms underlying CT45A1‐mediated aggressive phenotypes in MSI‐H CRC patients. CIC, cell‐in‐cell and NK, natural killer. The image was created using BioRender.

In an attempt to examine the clinical relevance of CT45A1, we collected 34 MSI‐H CRC specimens to quantify MLC2 activity and clinicopathological features associated with CT45A1 expression. A positive association between CT45A1 expression and MLC2 phosphorylation was observed (Fig. 6A,B). Homotypic CIC structures composed of two cancer cells with outer crescent nuclei and an inner cell encapsulated within a vacuole were observed in MSI‐H CRC tumors (Fig. 6C, red circles). However, while neither CT45A1 expression nor MLC2 phosphorylation was associated with the presence of a homotypic CIC structure (Fig. S9A,B and Fig. 6D), CT45A1 immunoreactivity‐positive cells in CIC structures were mainly the moon‐shaped outer cells (Fig. 6E, red arrows) in the CIC structure of MSI‐H CRC tumors (Fig. 6F). MSI‐H CRC patients whose survival data are available were further analyzed, and we found that patients with CT45A1‐positive tumors also exhibited worse survival outcomes compared to patients with CT45A1‐negative tumors (Fig. 6G).

## 4 Discussion

The cancer/testis antigen 45 (CT45) family comprises protein paralogs with high sequence conservation and structural similarity. CT45 expression is normally restricted to the testis. However, upregulated CT45 expression has been observed in many cancers and is associated with poor survival outcomes [30, 31, 32, 33, 34, 35]. Mechanistically, CT45A1 activates the ERK and CREB signaling pathways and promotes invasion in breast cancer [36] and lung cancer [37]. CT45A1 interacts with the TCF4/β‐catenin complex to promote the metastasis of osteosarcoma cell lines [38]. However, CT45 overexpression in advanced ovarian cancer cells reduces colony formation in the presence of carboplatin by inhibiting PP4 activity, and CT45 functions as a tumor antigen that activates cytotoxic T cells [39], suggesting the distinct functionality of CT45 proteins in specific TMEs.

Here, we demonstrated the unique functions of CT45A1 in CRC with different molecular characteristics. Overexpression of CT45A1 increased cancer cell resistance to NK cell killing via the RHO‐ROCK/MLCK‐MLC2 signaling axis (Fig. 4C–L) and promoted outer cell fate in homotypic CIC structures (Fig. 4M,N) without affecting cancer cell motility in MSI‐H CRC cell lines (Fig. 2C,D). In contrast, ectopic CT45A1 expression in MSS cell lines was associated with increased RAC1‐GTP levels (Fig. 5G), enhanced migratory potential (Fig. 5D,E), and an inner cell fate of homotypic CIC structures (Fig. 5I,J) without affecting NK cell susceptibility (Fig. 5H). Despite the differences at the cellular level, both CT45A1‐positive MSI‐H CRC patients (Fig. 6G) and CT45A1‐positive MSS CRC patients (Fig. S9C,D) had worse survival outcomes, suggesting the presence of distinct mechanisms underlying CT45A1‐driven cancer malignancy in these two CRC subtypes. Nuclear localization of CT45A1 was noted in CRC cells and tissues (Fig. S8A and Fig. 6A, respectively). Nuclear CT45A1 may interact with epigenetic modifiers to modulate the histone configuration to influence the RHO‐GTP/RAC1‐GTP levels indirectly in an MSS/MSI‐H context‐dependent manner. Understanding the genome‐wide CT45A1 occupancy at the single‐cell level will help decipher the transcriptional regulome in MSS/MSI‐H CRC patients.

Disruption of cytoskeleton dynamics is considered a treatment strategy for cancer metastasis [40]. ROCK plays a role in cell migration by regulating MLC protein phosphorylation. ROCK and MLCK regulate MLC phosphorylation directly or indirectly [41]. An increase in MLC phosphorylation leads to actin reorganization and stress fiber formation, enhancing cell invasiveness [42, 43]. Thus, disrupting ROCK and MLCK signaling represents a promising pharmacological strategy for treating metastatic disease. In this study, we demonstrated that inactivation of the RHO‐ROCK/MLCK‐MLC2 pathway by the ROCK inhibitor Y‐37632, the MLCK inhibitor ML‐7, or *MYLK* silencing counteracted CT45A1‐driven NK cell resistance (Fig. 4H,J,L and Fig. S10), suggesting a potential role of dephosphorylating MLC2 as an immune sensitizer. The inactivation of ROCK activity can further increase NK cell cytotoxicity by restoring PI3K‐dependent Akt activation [44]. Thus, clinical grade ROCK‐inhibiting agents may be applied in the regional MSI‐H TME along with ICBT to enhance the cytotoxic efficacy and lower the cost and treatment frequency of adoptive NK cell transfer therapy in aggressive CT45A1(+) MSI‐H CRC patients.

The CIC structure is generated by outer and inner cells of diverse cell types. Epithelial, mesenchymal, and neural cells, as well as megakaryocytes, can serve as outer cells, but the inner cells are mainly leukocytes [45]. The most commonly reported heterotypic CIC structure is between neutrophils and megakaryocytes in the bone marrow of experimental animals under sublethal irradiation [46] and blood loss [47]. In our study, increased heterotypic CIC structures of NK‐92MI cells and CT45A1‐expressing MSI‐H CRC cells were observed (Fig. S4A,B), and CT45A1‐expressing cells exhibited survival advantage in heterotypic CIC structures (Fig. S4C). As the internalization of NK cells by cancer cells enhances anticancer drug resistance in lung cancer [48], we cannot exclude the possibility that NK cell internalization enhances resistance to NK cell killing in CT45A1‐expressing MSI‐H CRC cells during the cytotoxic assay. CT45A1‐expressing MSI‐H CRC cells may also reduce the accessibility of CT45A1^Low^ CRC cells for NK cells spatially by uptaking NK cells to confer immune escape in a confined TME.

Homotypic CIC structures occur between cancer cells and are frequently reported in ascites [49, 50] and malignant effusion [51]. The presence of homotypic CIC structures in solid tumors is considered an indicator of poor prognosis for many cancers [52, 53, 54, 55]. CIC structures can be derived from cell cannibalism or entosis via different molecular mechanisms. In cell cannibalism, cancer cells engulf live and dead cells to scavenge nutrients to survive [56, 57]. During entosis, invading inner cells accumulate actin and myosin at the cell cortex, opposite the cell adhesions forming at the cell interface with outer cells, resulting in mechanical tension that drives the entotic invasion [58]. Increased cell tension regulated by the RHO–ROCK–actomyosin axis in the inner cell has been known to drive CIC formation following the establishment of cell–cell adhesion [59]. In contrast, oncogenic KRAS mutations activate RAC1 to promote cell deformability and allow the internalization of neighboring cells into outer cells [60].

Here, unexpectedly, we found that RHO‐activated MSI‐H CT45A1‐expressing cells acquired outer cell fates (Fig. 3D,E), while RAC1‐activated MSS CT45A1‐expressing cells were more likely to be the inner cells (Fig. 5I,J). Inhibiting MLC2 phosphorylation by silencing MYLK expression reduced the G(R) CIC subtype (Fig. 4M,N) in MSI‐H CRC cells, suggesting that the mechanism for CIC structure formation varies from classic entotic signaling under specific molecular features (i.e., MSS/MSI status). Although a low frequency of CIC structures (1–2%) was observed in MSI‐H CRC cells (Fig. S5B), the outer NK cell‐resistant, CT45A1‐expressing cancer cells in established homotypic CIC structures prevented the targeting of antibodies to inner cancer cells (Fig. 3K), indicating the formation of homotypic CIC structures may reduce the therapeutic efficacy of antibody‐conjugated oligonucleotides (ACOs), antibody‐conjugated drugs (ACDs), or ADCC. A booster antibody therapy or adoptive T/NK cell transfer may also be required and optimized for eradicating inner cancer cells. Nevertheless, the reduced G(R) CIC subtype (Fig. 3G) observed upon anti‐PD‐L1 antibody treatment, mainly used in MSI‐H CRC patients, may potentially improve the cancer immunotherapy by promoting the accessibility of inner cancer cells for NK cells and cytotoxic T cells.

The examination of CIC structures during routine histological checkups may aid the design of therapeutic interventions. Single‐clone expansion and single‐cell omics also may help decipher subclonal characterization and surface biomarker identification for isolating the MSS/MSI‐H CT45A1‐expressing subclones responsible for CIC structure formation. Endogenous CT45A1^High^ MSS/MSI‐H tumor organoids could be used in future studies to explore the molecular function of CT45A1 through a loss‐of‐function approach along with autologous or allogenic NK cells to optimize the treatment regimen.

## 5 Conclusions

In summary, this study is the first to compare the biological and clinical relevance of CT45A1 in MSI‐H and MSS CRC. We confirmed that, specifically in MSI‐H CRC cells, CT45A1 expression drives RHO‐ROCK/MLCK‐MLC2 signaling to enhance cancer cell resistance to NK cell cytotoxicity and generates a protective CIC structure, with CT45A1‐expressing cells becoming outer cells shielding the inner cancer cells from targeted antibody therapy and potentially subsequent antitumor immunity. Furthermore, the outer cell fate of CT45A1 (+) cells in a CIC structure was observed in MSI‐H CRC tumor sections, and MSI‐H CRC patients with upregulated CT45A1 expression exhibited increased MLC2 phosphorylation and poor clinical outcomes. Targeted inactivation of RHO‐ROCK/MLCK‐MLC2 signaling in CT45A1^High^ MSI‐H CRC cells may sensitize outer cancer cells to NK cell killing and enhance the accessibility of inner CT45A1^Low^ cancer cells for NK cells, benefiting cancer immunotherapy in MSI‐H CRC patients (Fig. 6H).

## Conflict of interest

The authors declare no conflict of interest.

## Author contributions

H‐WT, C‐CL, Y‐YL, and W‐LH initiated this study and directed the research. H‐YH, W‐CL, and H‐YL acquired and analyzed the data with the support of Y‐CT, Y‐YL, and W‐HY. H‐WT, H‐YH, C‐CL, Y‐YL, and W‐LH acquired the human specimens and performed clinicopathological analysis. H‐WT, H‐YH, C‐CL, Y‐YL, and W‐LH wrote and revised the manuscript.

### Peer review

The peer review history for this article is available at https://www.webofscience.com/api/gateway/wos/peer‐review/10.1002/1878‐0261.13736.

## Supporting information


**Fig. S1.** CT45A1 overexpression does not affect clonogenicity or malignant gene expression in MSI‐H CRC cells. (A) Histograms showing the viability of CRC cells at 48 h by MTT assay. *n* = 3. (B) Histograms showing the relative clonogenicity of CRC cells. *n* = 3. (C‐D) RT‐qPCR analysis showing the expression of EMT‐related genes (C) and stemness genes (D). *n* = 3. (E) Immunoblots showing the expression of pro‐caspase‐3 and cleaved caspase‐3 in CRC cells cultured with NK‐92MI cells for 90 min. MW, molecular weight. *n* = 2. The data are presented as the means ± SDs, and p values were determined by Student's t‐test for (A), (B), (C), and (D) or Mann–Whitney *U* test for (C, CDH2). *P < 0.05; **P < 0.01. ns, nonsignificant.

**Fig. S2.** Expression of NK cell inhibitory ligands on CRC cells. (A, C, E, G) Dot plots of the flow cytometry results for MHC‐I (A), PD‐L1 (C), ligands for Siglec‐7 (E), and ligands for Siglec‐9 (G). *n* = 1. (B, D, F, H) Histograms showing the percentages of MHC‐I (+) cells (B), PD‐L1 (+) cells (D), Siglec‐7 ligand (+) cells (F) and Siglec‐9 ligand (+) cells (H). *n* = 3 (B, right panel), (D), (F), and (H). *n* = 4 (B, left panel). The data are presented as the means ± SEMs, and p values were determined by Student's t‐test (B, right panel), (D), (F, right panel), and (H) or the Mann–Whitney *U* test for (B, left panel) and (F, left panel). ns, nonsignificant.

**Fig. S3.** Impact of CT45A1‐expressing CRC cells on NK‐92MI cells. (A) Dot plots showing the flow cytometry results for CD107a expression in NK‐92MI cells treated with the indicated CRC cells for 90 min. *n* = 1. (B‐C) Histograms showing the percentage of CD107a‐expressing DLD‐1 (B) and HCT‐15 (C) cell‐educated NK‐92MI cells. *n* = 3. The data are presented as the means ± SEMs, and p values were determined by Student's t‐test for (B) and (C). *P < 0.05; **P < 0.01; **P < 0.01. ns, nonsignificant.

**Fig. S4.** Heterotypic CIC structures generated by NK‐92MI cells and CRC cells. (A) Representative images of heterotypic CIC structure formation by unlabeled NK‐92MI cells and Venus‐carrying CT45A1‐expressing cancer cells or mCherry‐carrying vector cancer cells one hour after the seeding of NK‐92MI cells. White arrow, a heterotypic CIC structure; blue arrow, contact of NK‐92MI cells and CRC cells. Scale bar = 10 μm. (B) Histograms showing the percentage of heterotypic CIC structures one hour after NK cell seeding. *n* = 3. (C) Percentage of the indicated cell events in CRC cells with homotypic structures under time‐lapse tracking (5 min per image for 5 h). There were 13 (DLD‐1‐VEC‐C2) and 25 (DLD‐1‐CT45A1‐V2) heterotypic CIC structures counted. *n* = 3. (D) Histograms showing the contact and in‐cell killing of NK‐92MI cells under time‐lapse tracking. There were 65 (DLD‐1‐VEC‐C2) and 64 (DLD‐1‐CT45A1‐V2) killing events counted. *n* = 3. The data are presented as the means ± SEMs, and p values were determined by Student's t‐test for (B), (C), and (D). *P < 0.05; **P < 0.01; **P < 0.01. ns, nonsignificant.

**Fig. S5.** Homotypic CIC structures generated by CRC cells. (A) Representative images showing the homotypic CIC structures generated by CT45A1‐expressing cancer cells marked with Venus expression (green) and vector cells labeled with mCherry expression (red). G(R) is a CIC structure with outer CT45A1‐expressing cells and inner vector cells; arrows indicate a CIC structure. Scale bar = 10 μm. (B) Histograms showing the CIC structure frequency of CRC cells in basal RPMI medium at 24 and 48 h. *n* = 3. (C‐D) Histograms showing cancer cell viability in basal RPMI medium at 24 h (C) and 48 h (D). Vec‐C2, vec‐mCherry‐expressing red cells; CT45A1‐V2, CT45A1‐Venus‐expressing green cells. *n* = 3. (E) Representative images showing the homotypic CIC structures generated by CT45A1‐expressing cancer cells marked with Venus expression (green) and vector‐expressing cells labeled with mCherry (red) in the presence of the indicated antibodies (100 μg/mL). Arrows indicate a CIC structure. Scale bar = 10 μm. (F) Western blots showing the expression of CT45A1 after anti‐PD‐L1 treatment (100 μg/mL) for 48 h. *n* = 2. The data represent the means ± SEMs for (B), and the data represent the means ± SDs for (C) and (D). p values were determined by Student's t‐test. *P < 0.05. ns, nonsignificant.

**Fig. S6.** Homotypic CIC structures protect inner CRC cells from antibody targeting. (A) Confocal images showing the targeting of antibodies to established homotypic CIC structures. Scale bar = 20 μm. (B‐C) Upper: Representative images showing the labeling of established CIC structures with anti‐EGFR (B) and anti‐PD‐L1 (C) antibodies. The dashed line arrow is the intensity path across a CIC structure used to quantify fluorescent signals (arrow, end of the path direction). Lower: Plots showing fluorescent intensities along the dashed arrow line (the left‐to‐right distance direction: star to the end arrow) in the upper right panel. Scale bar = 10 μm. *n* = 1.

**Fig. S7.** Role of MLCK activity in cancer cell vulnerability to NK cells in MSI‐H CRC cells. (A‐C) Western blots showing the levels of RHO‐GTP (A), RAC1‐GTP (B), and phosphorylated MLC2 (S19) (C) in CRC cells. Two biological replicates are shown. MW, molecular weight. *n* = 2. (D) Immunoblots of phosphorylated MLC2 (S19) after Y‐27632 and ML‐7 treatment for 24 h. *n* = 1. (E) Western blot images showing MLC2 (S19) phosphorylation in HCT15‐CT45A1 cells after ML‐7 treatment for 24 h. *n* = 1. (F) NK‐92MI cytotoxicity assay. CT45A1‐expressing HCT‐15 cells were treated with ML‐7 for 24 h, followed by NK92‐MI treatment 4 h before MTT analysis. The data are presented as the means ± SDs. The p value was determined by two‐way ANOVA followed by Tukey's *post hoc* test. *P < 0.05; **P < 0.01; ***P < 0.001. *n* = 4. (G) Western blots showing the expression of MLCK and phosphorylated MLC2 (S19) upon knocking down MYLK (*MLCK*) in DLD‐1‐CT45A1 cells. *n* = 1.

**Fig. S8.** Expression of RHO‐GTP and RAC1‐GTP in ectopic CT45A1‐expressing MSS CRC cells. (A) Confocal images showing the endogenous expression of CT45A1 in MSS (SW480 and HT29) and MSI‐H (DLD‐1 and HCT‐15) CRC cell lines. Scale bar = 20 μm. (B‐C) Western blots showing the levels of RHO‐GTP (B) and RAC1‐GTP (C) in HT29 cells. Two biological replicates are shown. MW, molecular weight. *n* = 2.

**Fig. S9.** Associations among CT45A1, MLC2 phosphorylation, and CIC structure in CRC patients. (A‐B) Tables summarizing the correlation of CIC structure positivity with immunoreactivity to CT45A1 and p‐MLC2 (S19) antibodies. Fisher's exact test was used to estimate the p values. (C) IHC images showing the immunoreactivity of CT45A1 in clinical CRC specimens. Scale bar = 200 μm. (D) Kaplan–Meier plot depicting the overall survival of 220 MSS CRC patients. The p values were estimated by log‐rank t tests.

**Fig. S10.** Uncropped western blot images. The uncropped blots and molecular weight labels are shown in the indicated images.

**Table S1.** Primer list for qPCR.

**Table S2.** Antibody list.

**Table S3.** List for the reagents and chemicals used in this study.

**Table S4.** The aggressive MSI‐H CRC signature.

**Table S5.** Upregulated CT45A1 signature in MSI‐H CRC cells.

**Table S6.** Downregulated CT45A1 signature in MSI‐H CRC cells.

## Data Availability

Data supporting the findings of this study are available within this article and supplementary materials. The RNA‐seq data have been deposited under the accession number GSE193554. Uncropped western blot images are shown in Fig. S10.
