# Supplementary figures and images for: CT45A1‐mediated MLC2 (MYL9) phosphorylation promotes natural killer cell resistance and outer cell fate in a cell‐in‐cell structure, potentiating the progression of microsatellite instability‐high colorectal cancer
Source: Mol Oncol. 2024 Sep 25;19(2):430–51. doi: 10.1002/1878-0261.13736 (PMC11793002; doi:10.1002/1878-0261.13736)

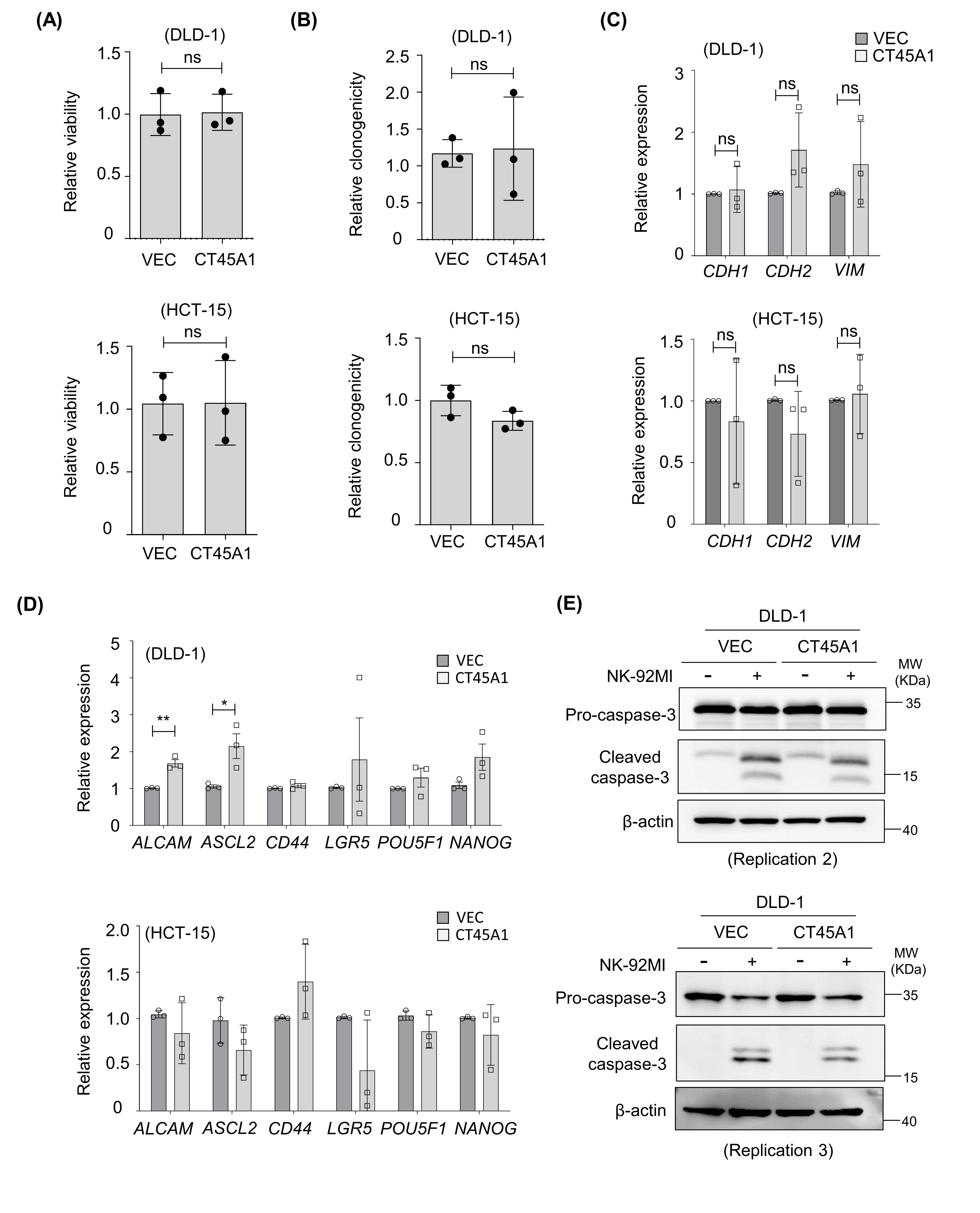

Supplement: Supplementary file 1 — Fig. S1. CT45A1 overexpression does not affect clonogenicity or malignant gene expression in MSI‐H CRC cells. (A) Histograms showing the viability of CRC cells at 48 h by MTT assay. n = 3. (B) Histograms showing the relative clonogenicity of CRC cells. n = 3. (C‐D) RT‐qPCR analysis showing the expression of EMT‐related genes (C) and stemness genes (D). n = 3. (E) Immunoblots showing the expression of pro‐caspase‐3 and cleaved caspase‐3 in CRC cells cultured with NK‐92MI cells for 90 min. MW, molecular weight. n = 2. The data are presented as the means ± SDs, and p values were determined by Student's t‐test for (A), (B), (C), and (D) or Mann–Whitney U test for (C, CDH2). *P < 0.05; **P < 0.01. ns, nonsignificant. [file MOL2-19-430-s008.tif]

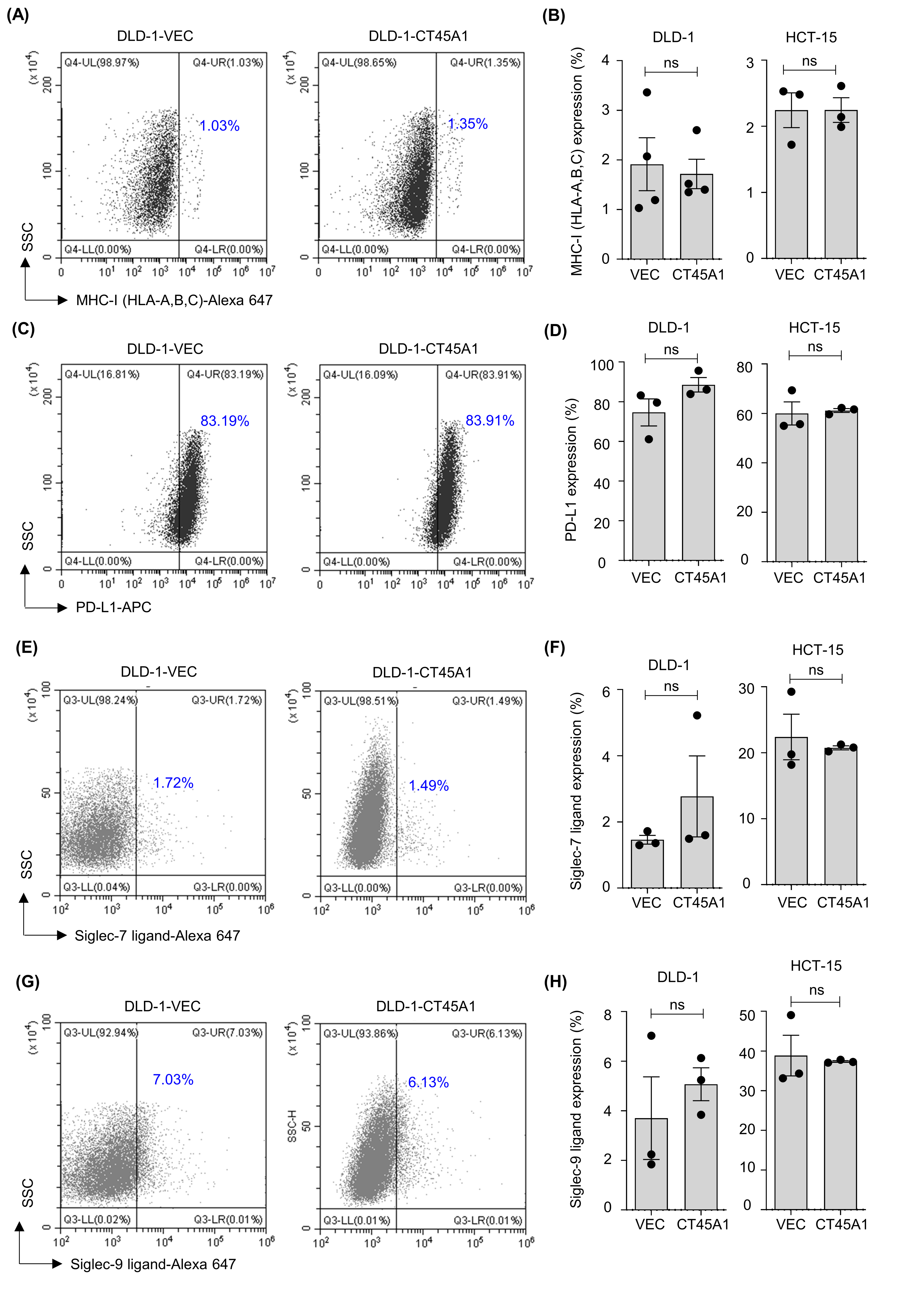

Supplement: Supplementary file 2 — Fig. S2. Expression of NK cell inhibitory ligands on CRC cells. (A, C, E, G) Dot plots of the flow cytometry results for MHC‐I (A), PD‐L1 (C), ligands for Siglec‐7 (E), and ligands for Siglec‐9 (G). n = 1. (B, D, F, H) Histograms showing the percentages of MHC‐I (+) cells (B), PD‐L1 (+) cells (D), Siglec‐7 ligand (+) cells (F) and Siglec‐9 ligand (+) cells (H). n = 3 (B, right panel), (D), (F), and (H). n = 4 (B, left panel). The data are presented as the means ± SEMs, and p values were determined by Student's t‐test (B, right panel), (D), (F, right panel), and (H) or the Mann–Whitney U test for (B, left panel) and (F, left panel). ns, nonsignificant. [file MOL2-19-430-s007.tif]

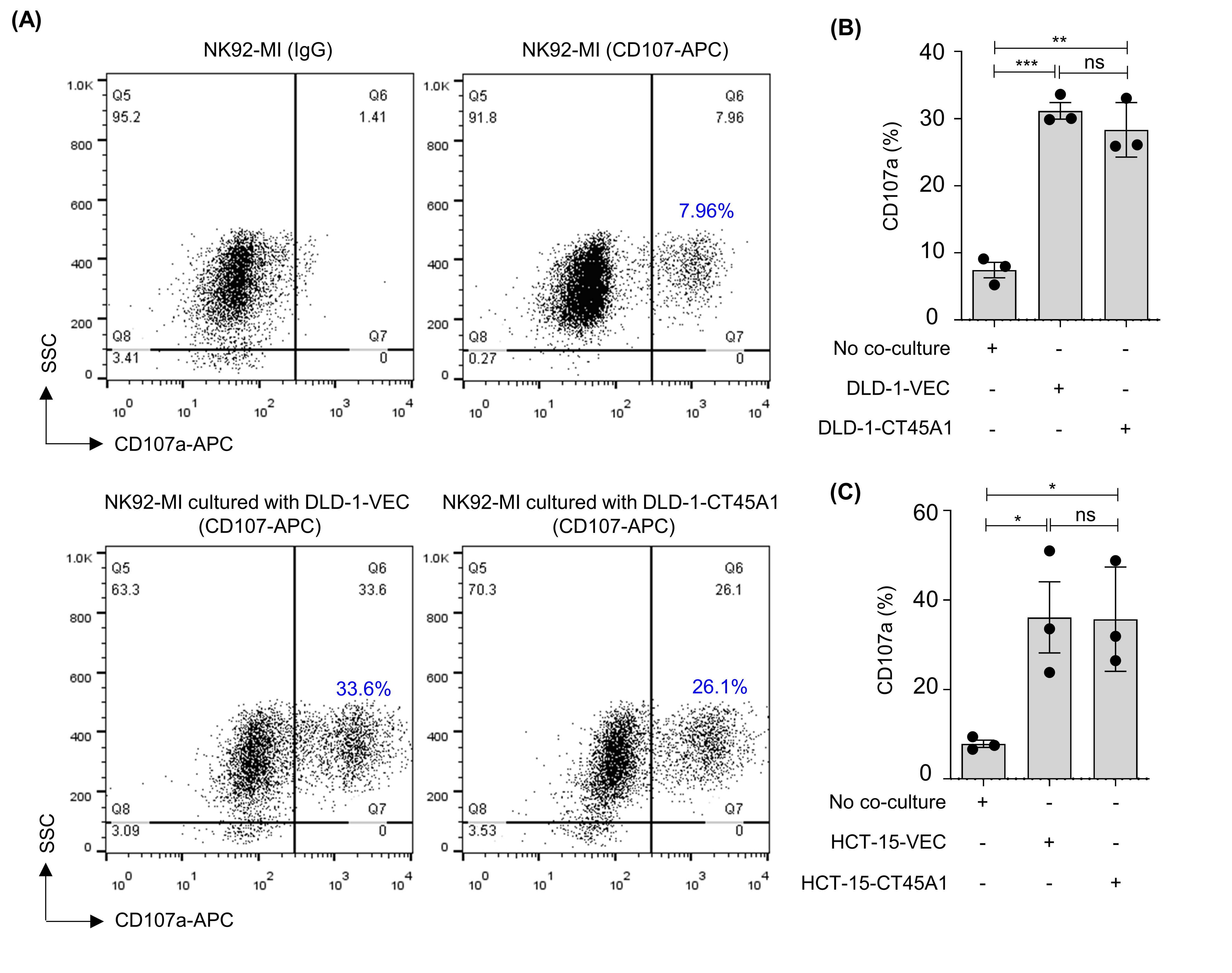

Supplement: Supplementary file 3 — Fig. S3. Impact of CT45A1‐expressing CRC cells on NK‐92MI cells. (A) Dot plots showing the flow cytometry results for CD107a expression in NK‐92MI cells treated with the indicated CRC cells for 90 min. n = 1. (B‐C) Histograms showing the percentage of CD107a‐expressing DLD‐1 (B) and HCT‐15 (C) cell‐educated NK‐92MI cells. n = 3. The data are presented as the means ± SEMs, and p values were determined by Student's t‐test for (B) and (C). *P < 0.05; **P < 0.01; **P < 0.01. ns, nonsignificant. [file MOL2-19-430-s001.tif]

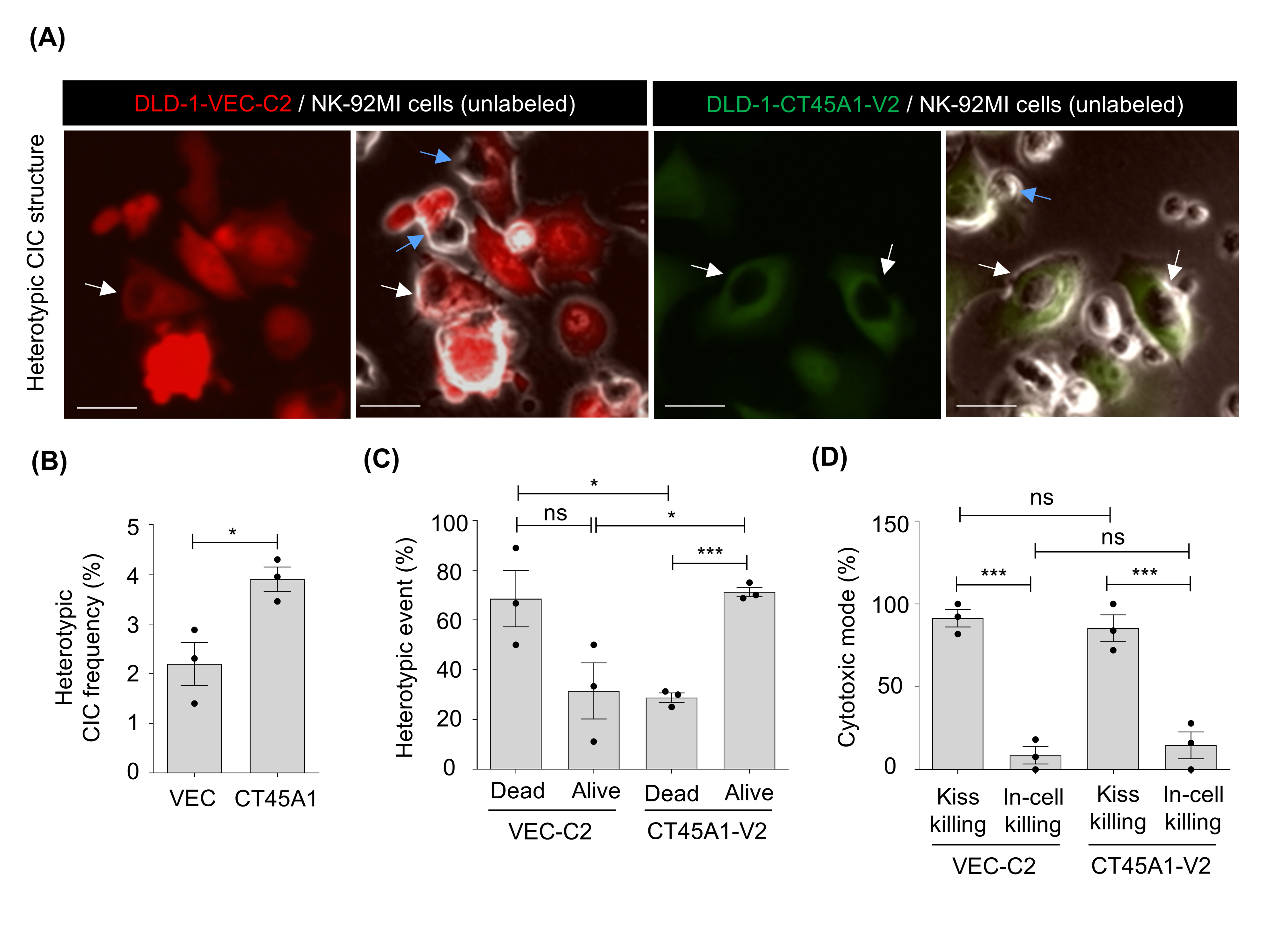

Supplement: Supplementary file 4 — Fig. S4. Heterotypic CIC structures generated by NK‐92MI cells and CRC cells. (A) Representative images of heterotypic CIC structure formation by unlabeled NK‐92MI cells and Venus‐carrying CT45A1‐expressing cancer cells or mCherry‐carrying vector cancer cells one hour after the seeding of NK‐92MI cells. White arrow, a heterotypic CIC structure; blue arrow, contact of NK‐92MI cells and CRC cells. Scale bar = 10 μm. (B) Histograms showing the percentage of heterotypic CIC structures one hour after NK cell seeding. n = 3. (C) Percentage of the indicated cell events in CRC cells with homotypic structures under time‐lapse tracking (5 min per image for 5 h). There were 13 (DLD‐1‐VEC‐C2) and 25 (DLD‐1‐CT45A1‐V2) heterotypic CIC structures counted. n = 3. (D) Histograms showing the contact and in‐cell killing of NK‐92MI cells under time‐lapse tracking. There were 65 (DLD‐1‐VEC‐C2) and 64 (DLD‐1‐CT45A1‐V2) killing events counted. n = 3. The data are presented as the means ± SEMs, and p values were determined by Student's t‐test for (B), (C), and (D). *P < 0.05; **P < 0.01; **P < 0.01. ns, nonsignificant. [file MOL2-19-430-s003.tif]

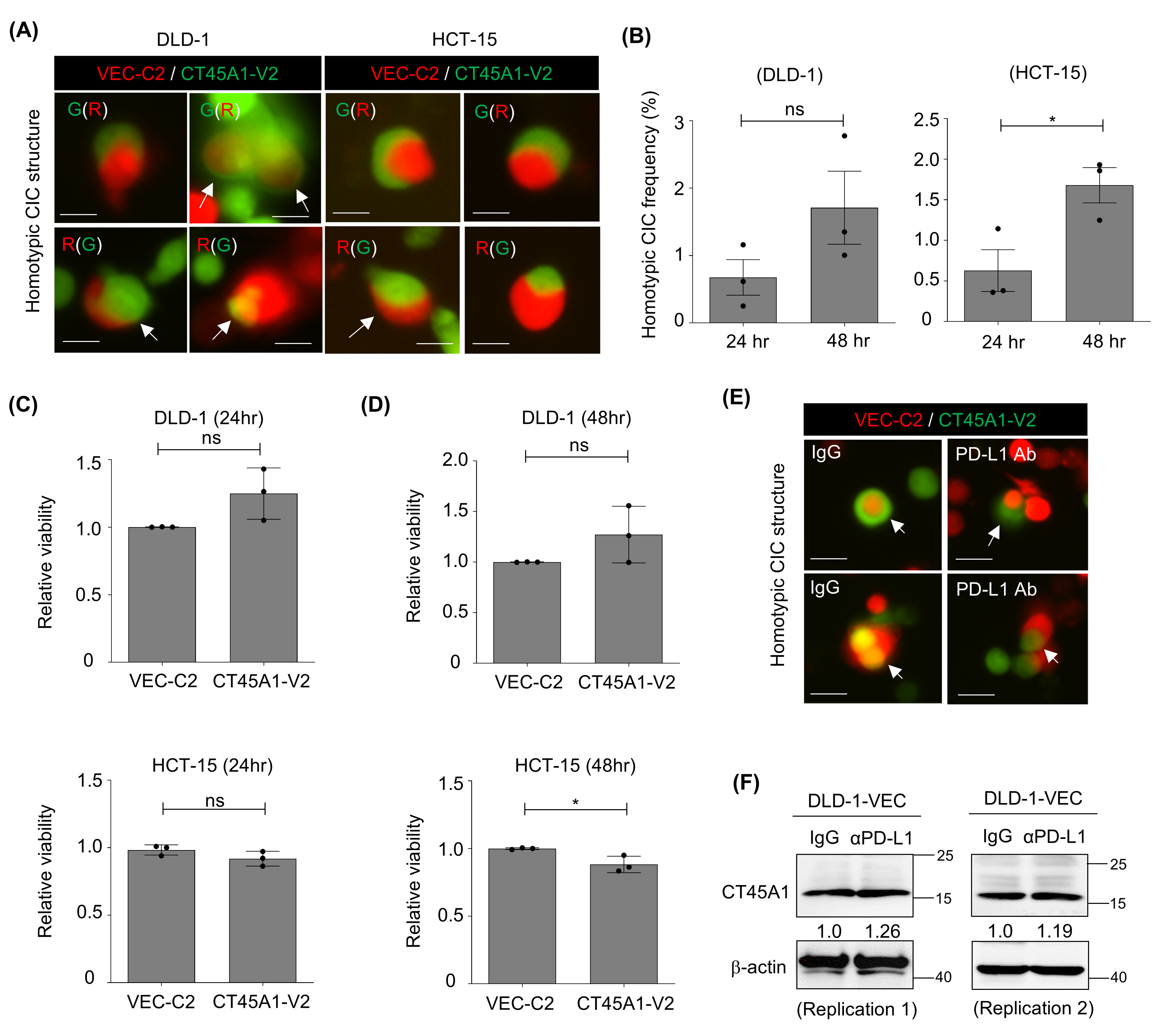

Supplement: Supplementary file 5 — Fig. S5. Homotypic CIC structures generated by CRC cells. (A) Representative images showing the homotypic CIC structures generated by CT45A1‐expressing cancer cells marked with Venus expression (green) and vector cells labeled with mCherry expression (red). G(R) is a CIC structure with outer CT45A1‐expressing cells and inner vector cells; arrows indicate a CIC structure. Scale bar = 10 μm. (B) Histograms showing the CIC structure frequency of CRC cells in basal RPMI medium at 24 and 48 h. n = 3. (C‐D) Histograms showing cancer cell viability in basal RPMI medium at 24 h (C) and 48 h (D). Vec‐C2, vec‐mCherry‐expressing red cells; CT45A1‐V2, CT45A1‐Venus‐expressing green cells. n = 3. (E) Representative images showing the homotypic CIC structures generated by CT45A1‐expressing cancer cells marked with Venus expression (green) and vector‐expressing cells labeled with mCherry (red) in the presence of the indicated antibodies (100 μg/mL). Arrows indicate a CIC structure. Scale bar = 10 μm. (F) Western blots showing the expression of CT45A1 after anti‐PD‐L1 treatment (100 μg/mL) for 48 h. n = 2. The data represent the means ± SEMs for (B), and the data represent the means ± SDs for (C) and (D). p values were determined by Student's t‐test. *P < 0.05. ns, nonsignificant. [file MOL2-19-430-s014.tif]

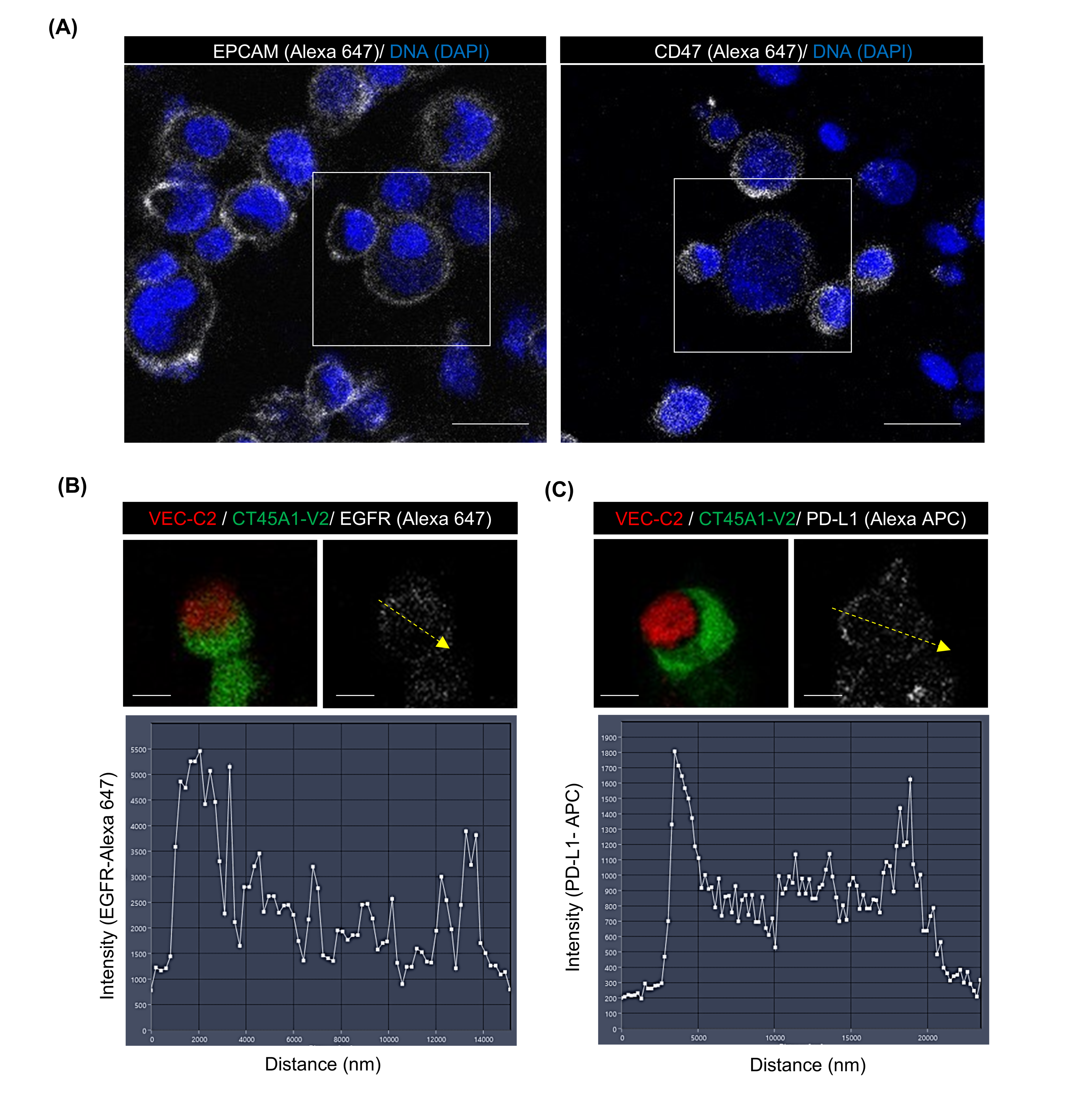

Supplement: Supplementary file 6 — Fig. S6. Homotypic CIC structures protect inner CRC cells from antibody targeting. (A) Confocal images showing the targeting of antibodies to established homotypic CIC structures. Scale bar = 20 μm. (B‐C) Upper: Representative images showing the labeling of established CIC structures with anti‐EGFR (B) and anti‐PD‐L1 (C) antibodies. The dashed line arrow is the intensity path across a CIC structure used to quantify fluorescent signals (arrow, end of the path direction). Lower: Plots showing fluorescent intensities along the dashed arrow line (the left‐to‐right distance direction: star to the end arrow) in the upper right panel. Scale bar = 10 μm. n = 1. [file MOL2-19-430-s011.tif]

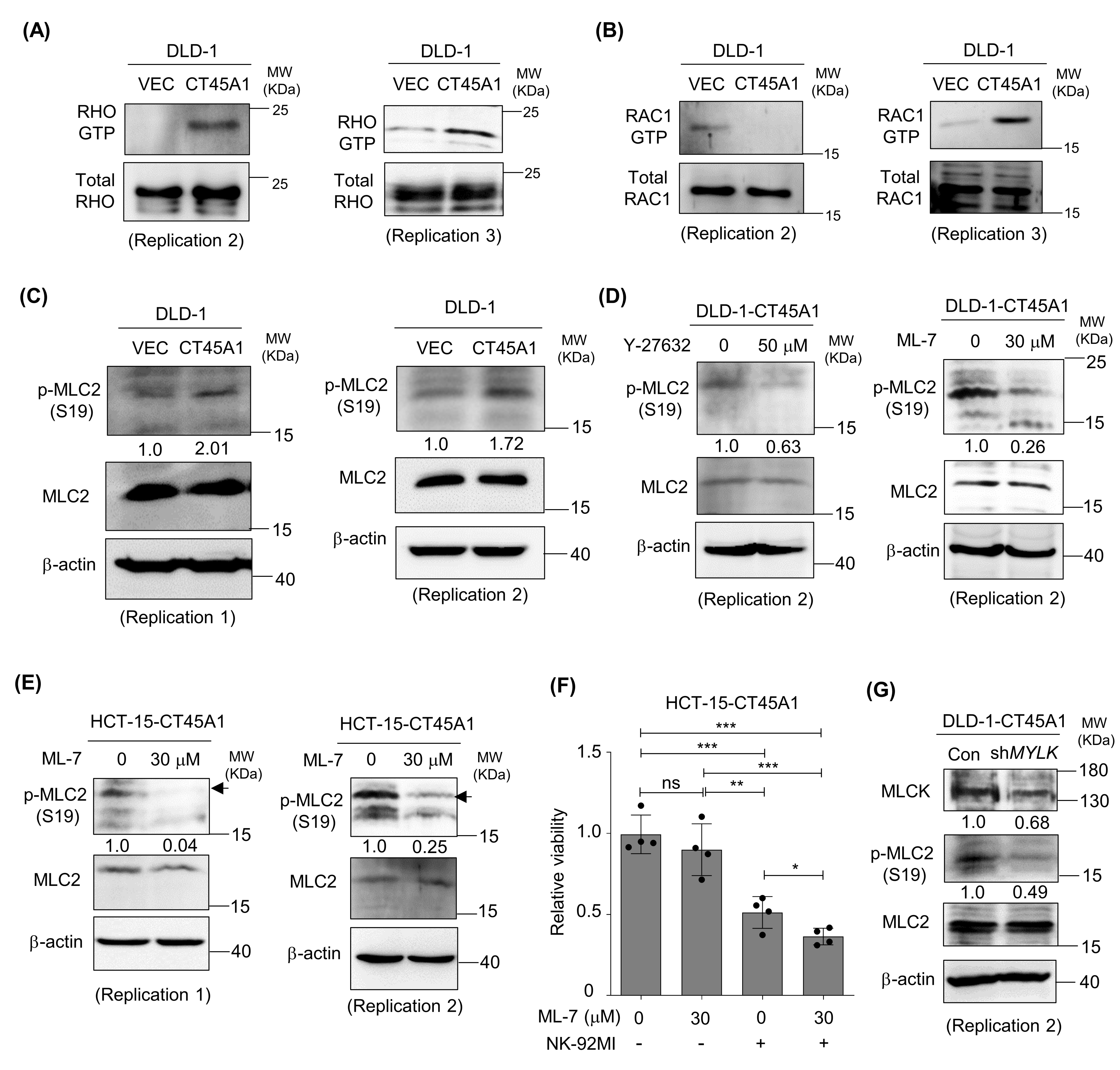

Supplement: Supplementary file 7 — Fig. S7. Role of MLCK activity in cancer cell vulnerability to NK cells in MSI‐H CRC cells. (A‐C) Western blots showing the levels of RHO‐GTP (A), RAC1‐GTP (B), and phosphorylated MLC2 (S19) (C) in CRC cells. Two biological replicates are shown. MW, molecular weight. n = 2. (D) Immunoblots of phosphorylated MLC2 (S19) after Y‐27632 and ML‐7 treatment for 24 h. n = 1. (E) Western blot images showing MLC2 (S19) phosphorylation in HCT15‐CT45A1 cells after ML‐7 treatment for 24 h. n = 1. (F) NK‐92MI cytotoxicity assay. CT45A1‐expressing HCT‐15 cells were treated with ML‐7 for 24 h, followed by NK92‐MI treatment 4 h before MTT analysis. The data are presented as the means ± SDs. The p value was determined by two‐way ANOVA followed by Tukey's post hoc test. *P < 0.05; **P < 0.01; ***P < 0.001. n = 4. (G) Western blots showing the expression of MLCK and phosphorylated MLC2 (S19) upon knocking down MYLK (MLCK) in DLD‐1‐CT45A1 cells. n = 1. [file MOL2-19-430-s006.tiff]

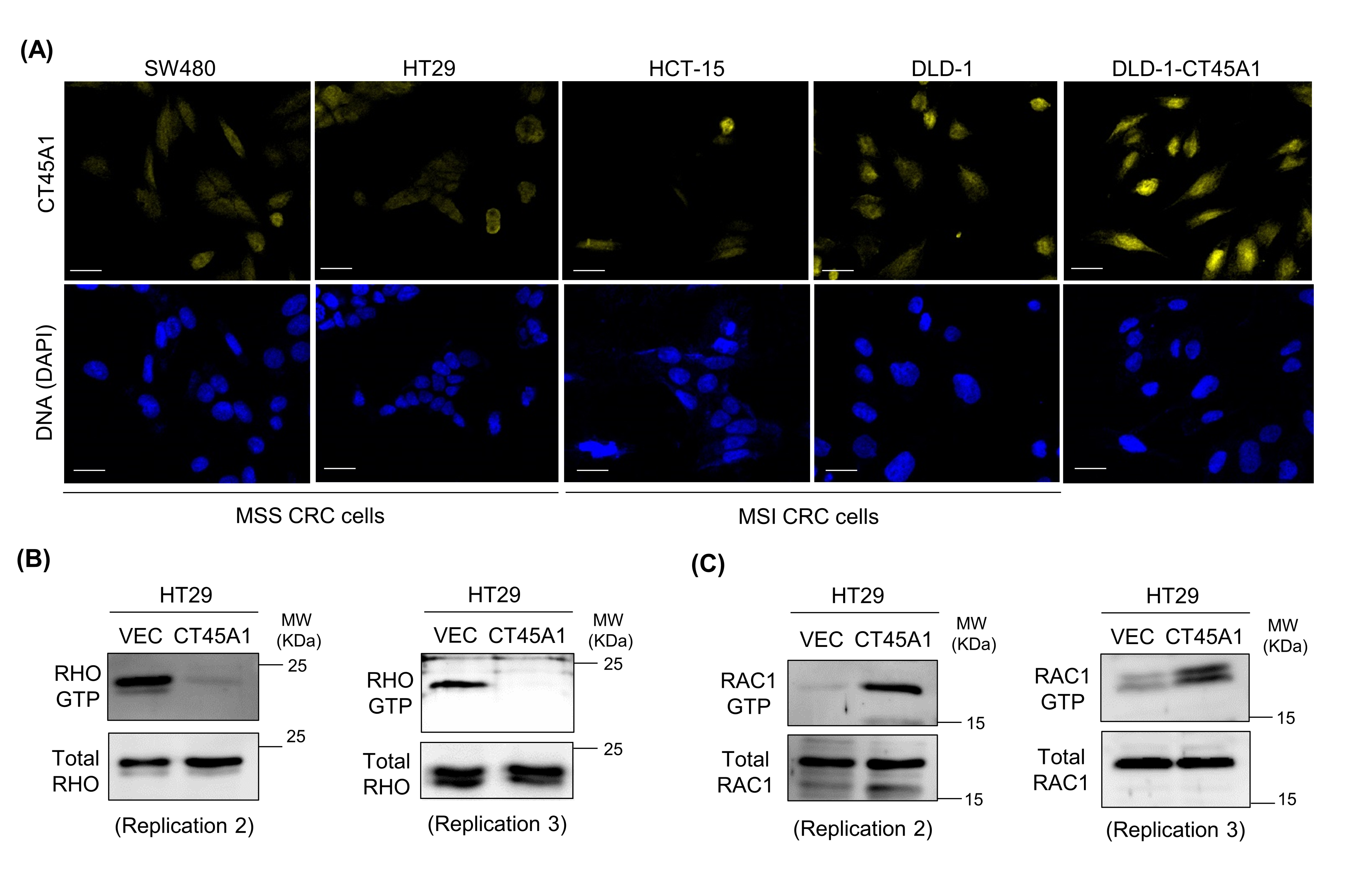

Supplement: Supplementary file 8 — Fig. S8. Expression of RHO‐GTP and RAC1‐GTP in ectopic CT45A1‐expressing MSS CRC cells. (A) Confocal images showing the endogenous expression of CT45A1 in MSS (SW480 and HT29) and MSI‐H (DLD‐1 and HCT‐15) CRC cell lines. Scale bar = 20 μm. (B‐C) Western blots showing the levels of RHO‐GTP (B) and RAC1‐GTP (C) in HT29 cells. Two biological replicates are shown. MW, molecular weight. n = 2. [file MOL2-19-430-s010.tif]

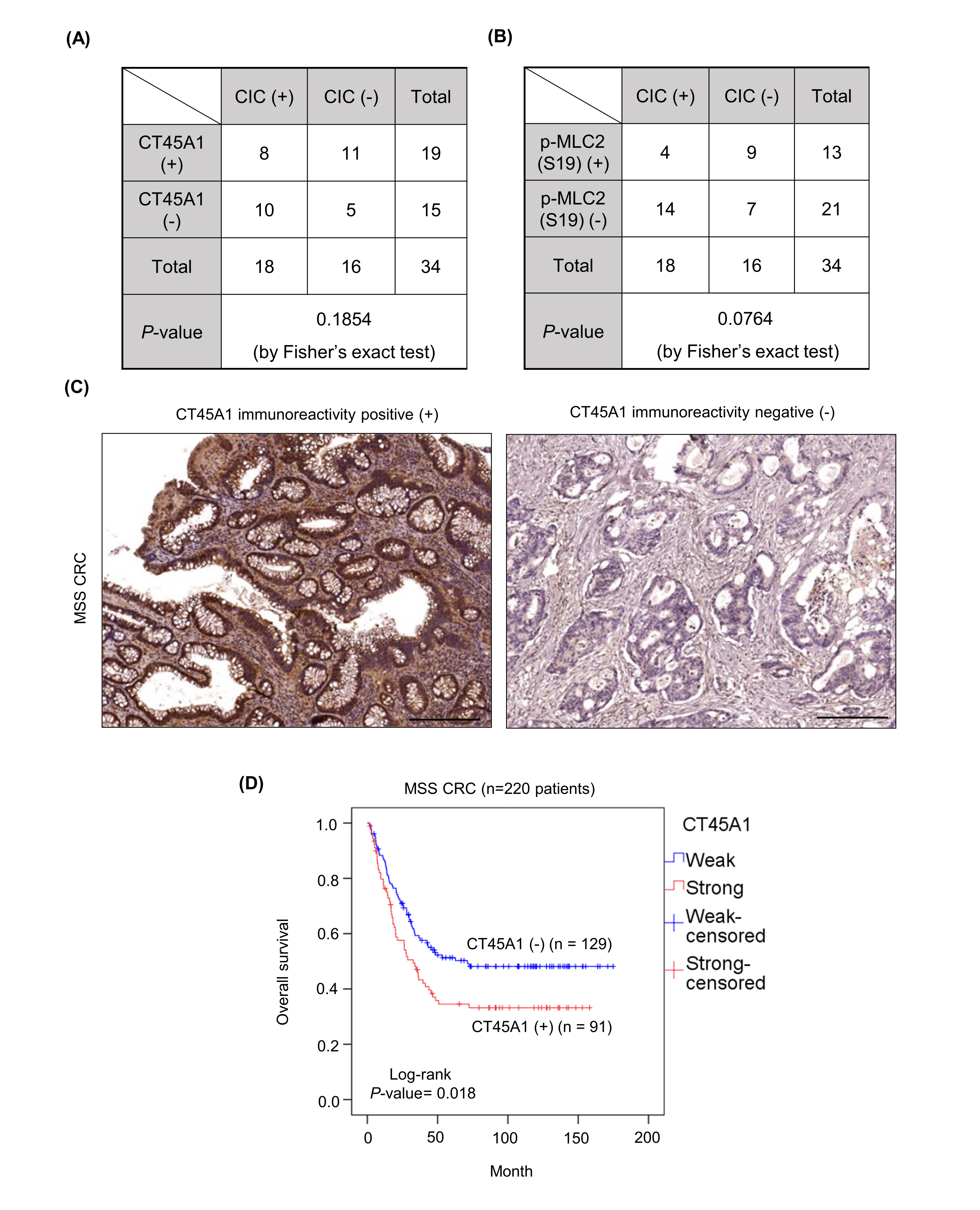

Supplement: Supplementary file 9 — Fig. S9. Associations among CT45A1, MLC2 phosphorylation, and CIC structure in CRC patients. (A‐B) Tables summarizing the correlation of CIC structure positivity with immunoreactivity to CT45A1 and p‐MLC2 (S19) antibodies. Fisher's exact test was used to estimate the p values. (C) IHC images showing the immunoreactivity of CT45A1 in clinical CRC specimens. Scale bar = 200 μm. (D) Kaplan–Meier plot depicting the overall survival of 220 MSS CRC patients. The p values were estimated by log‐rank t tests. [file MOL2-19-430-s016.tif]

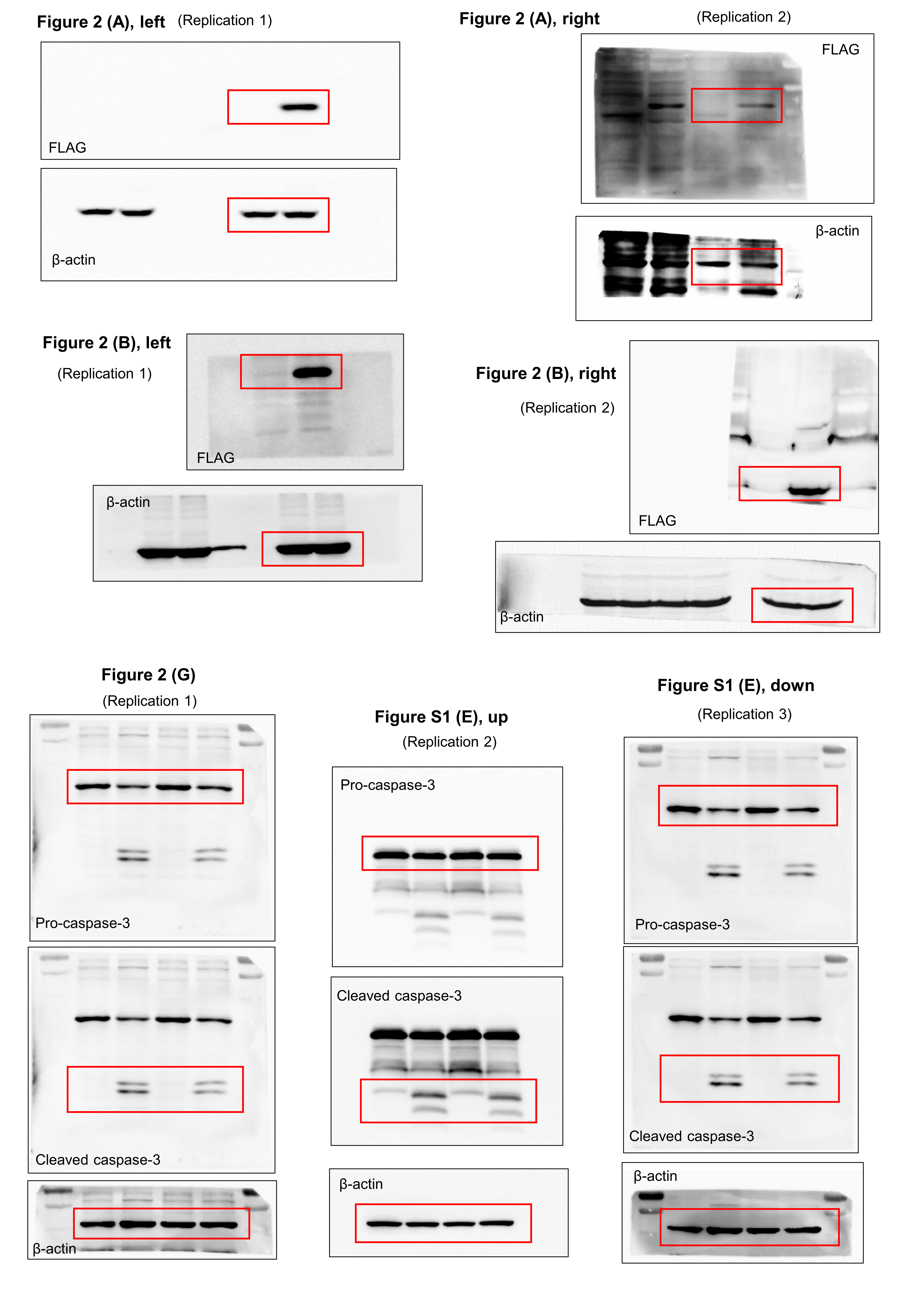

Supplement: Supplementary file 10 — Fig. S10. Uncropped western blot images. The uncropped blots and molecular weight labels are shown in the indicated images. [file MOL2-19-430-s012.zip › Figure S10-1.tif]

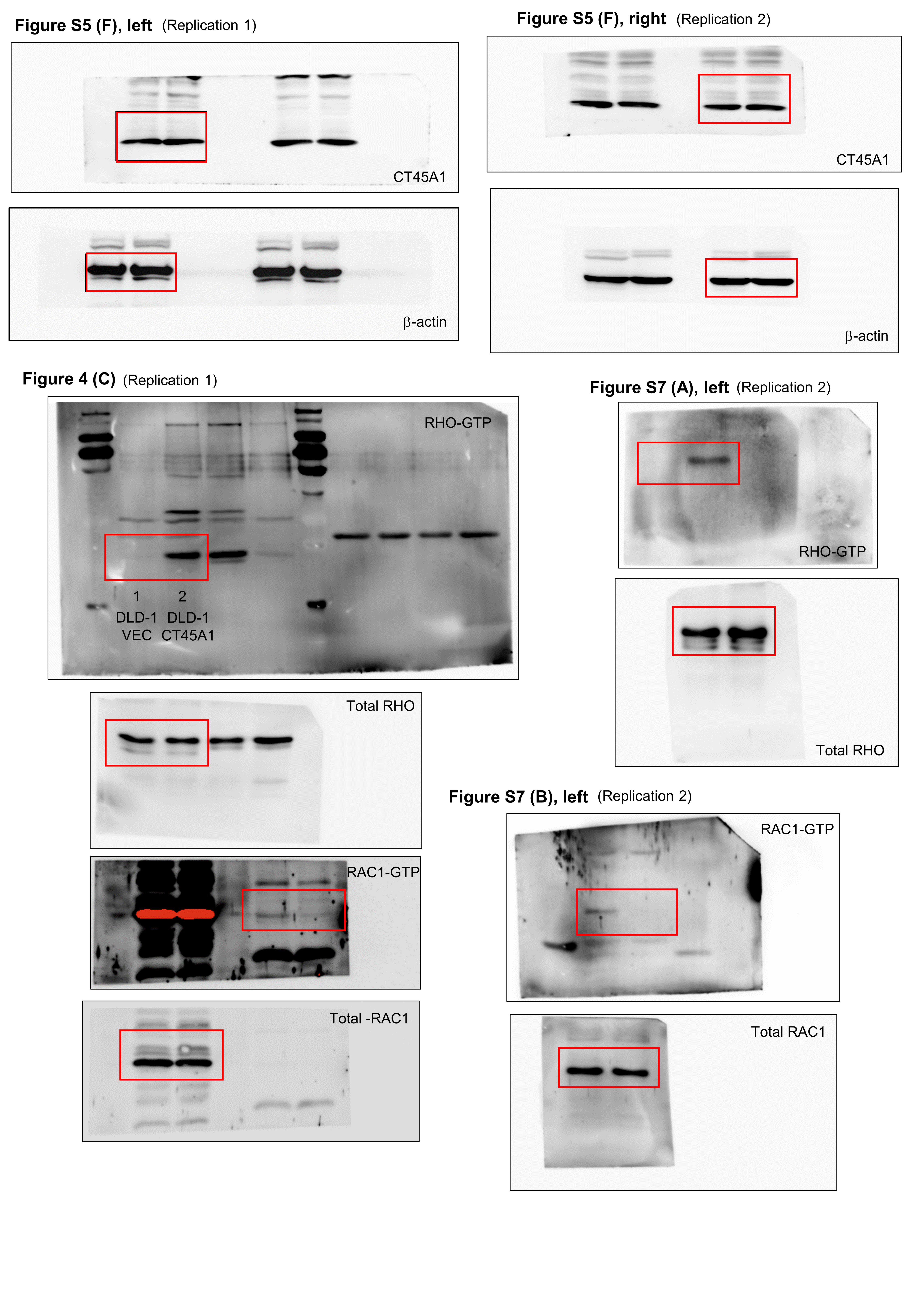

Supplement: Supplementary file 10 — Fig. S10. Uncropped western blot images. The uncropped blots and molecular weight labels are shown in the indicated images. [file MOL2-19-430-s012.zip › Figure S10-2.tif]

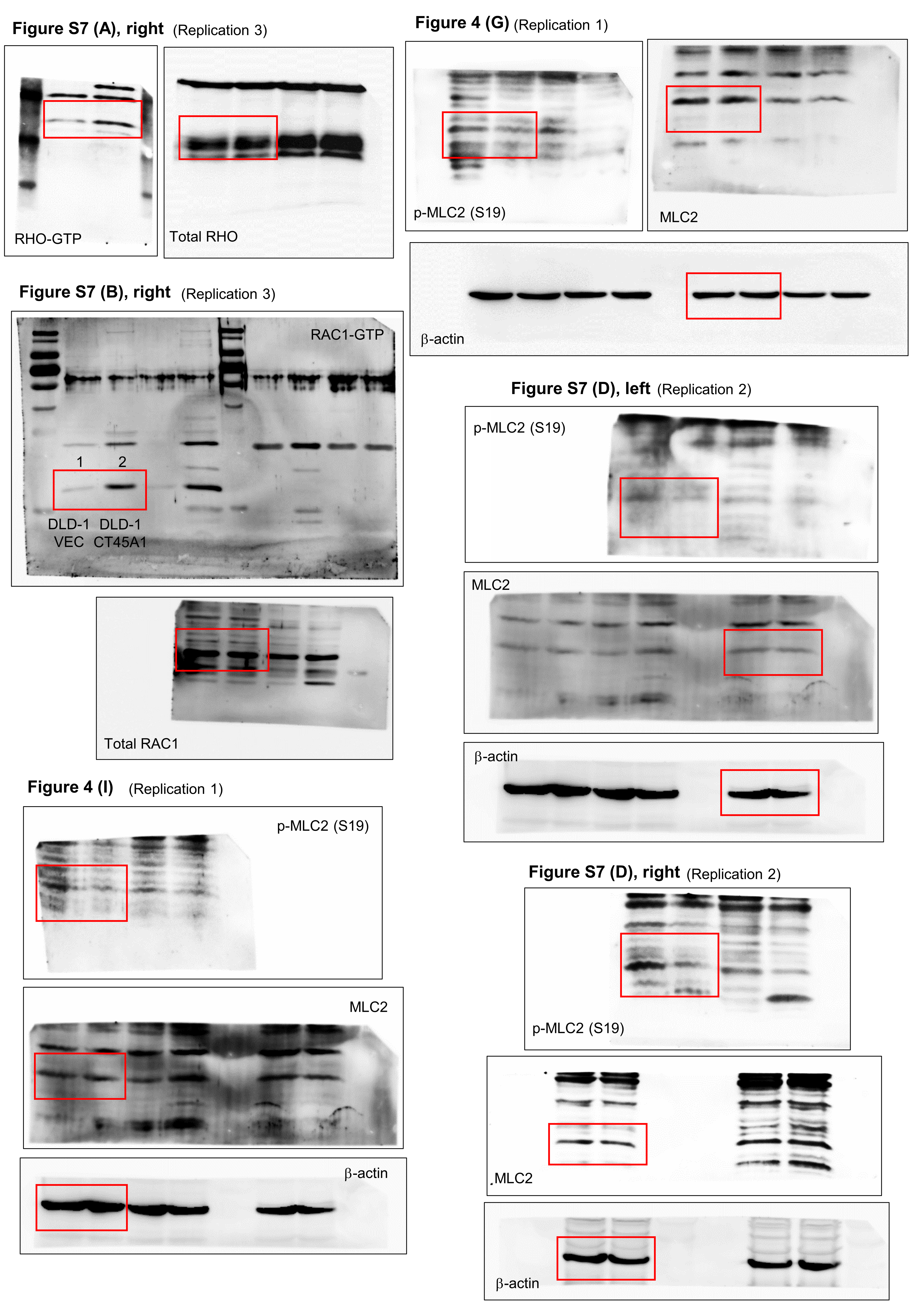

Supplement: Supplementary file 10 — Fig. S10. Uncropped western blot images. The uncropped blots and molecular weight labels are shown in the indicated images. [file MOL2-19-430-s012.zip › Figure S10-3.tif]

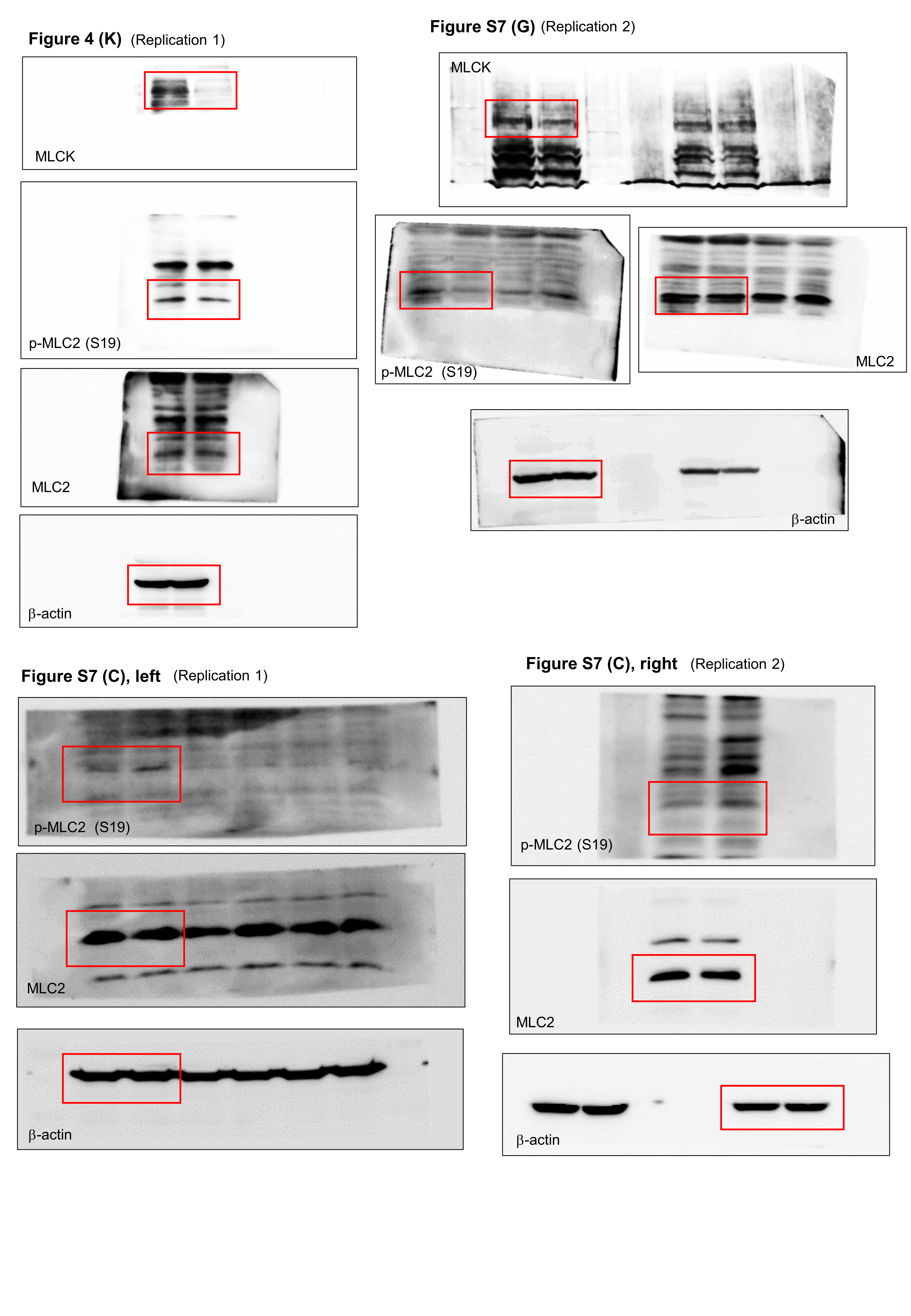

Supplement: Supplementary file 10 — Fig. S10. Uncropped western blot images. The uncropped blots and molecular weight labels are shown in the indicated images. [file MOL2-19-430-s012.zip › Figure S10-4.tif]

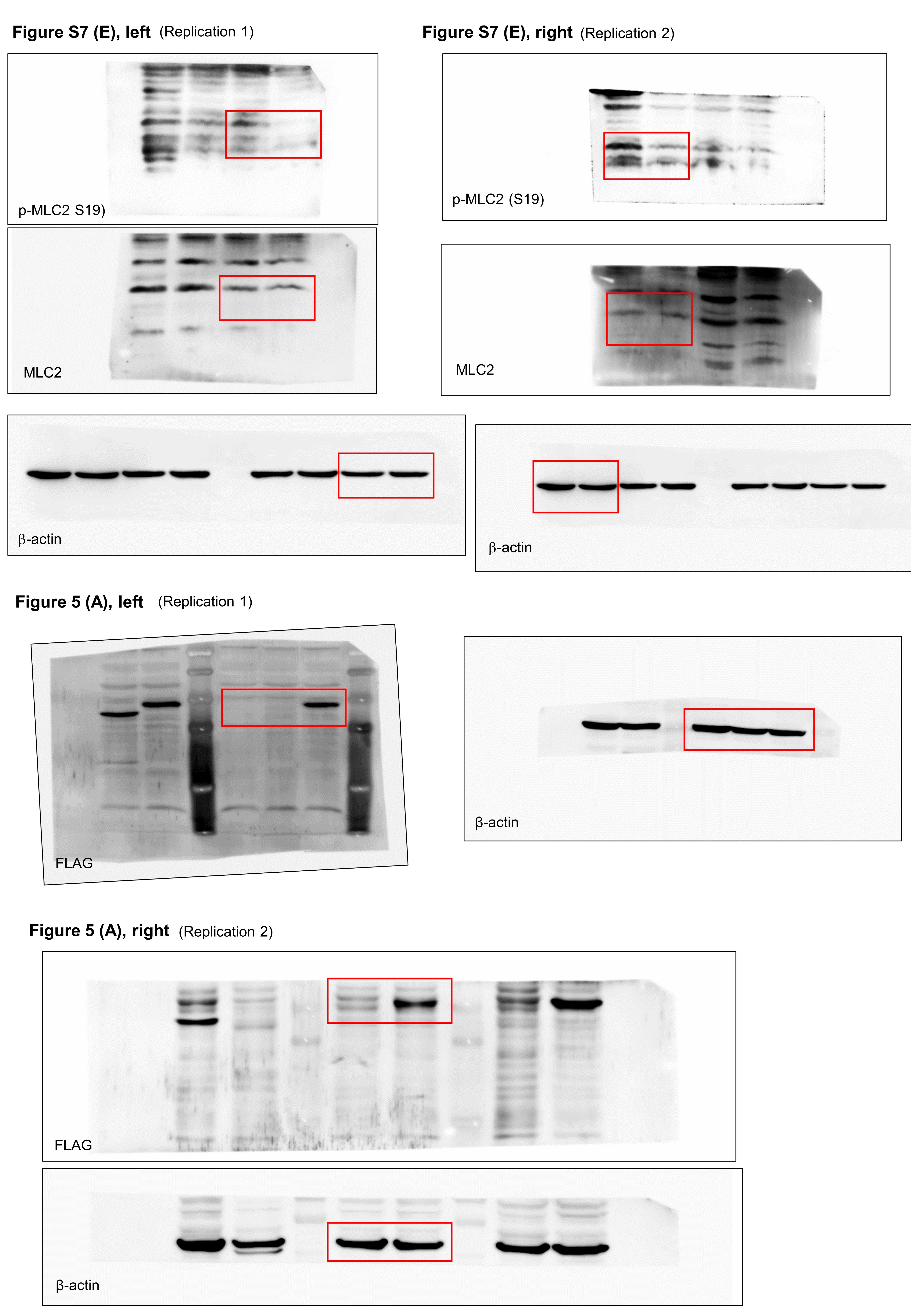

Supplement: Supplementary file 10 — Fig. S10. Uncropped western blot images. The uncropped blots and molecular weight labels are shown in the indicated images. [file MOL2-19-430-s012.zip › Figure S10-5.tif]

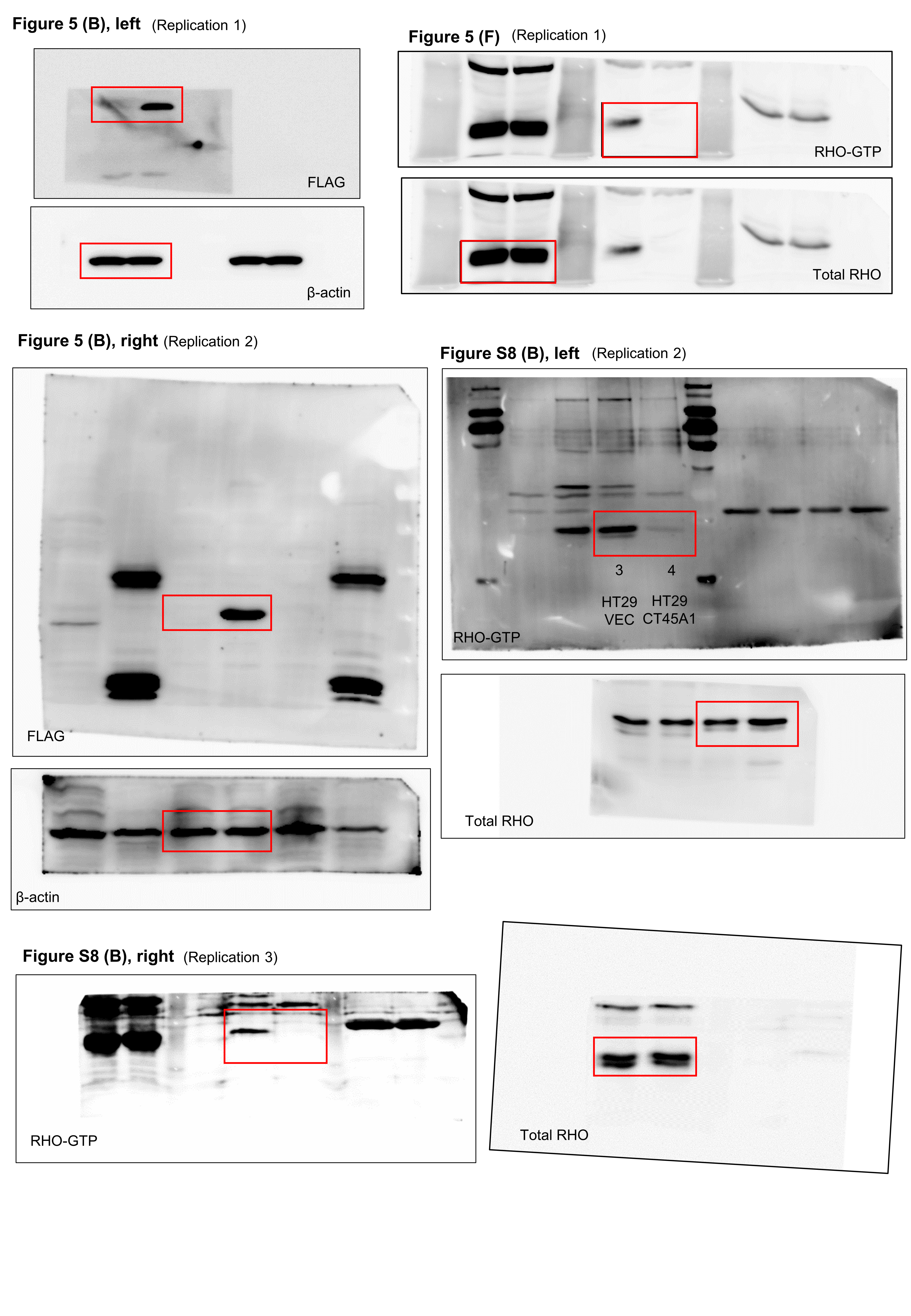

Supplement: Supplementary file 10 — Fig. S10. Uncropped western blot images. The uncropped blots and molecular weight labels are shown in the indicated images. [file MOL2-19-430-s012.zip › Figure S10-6.tif]

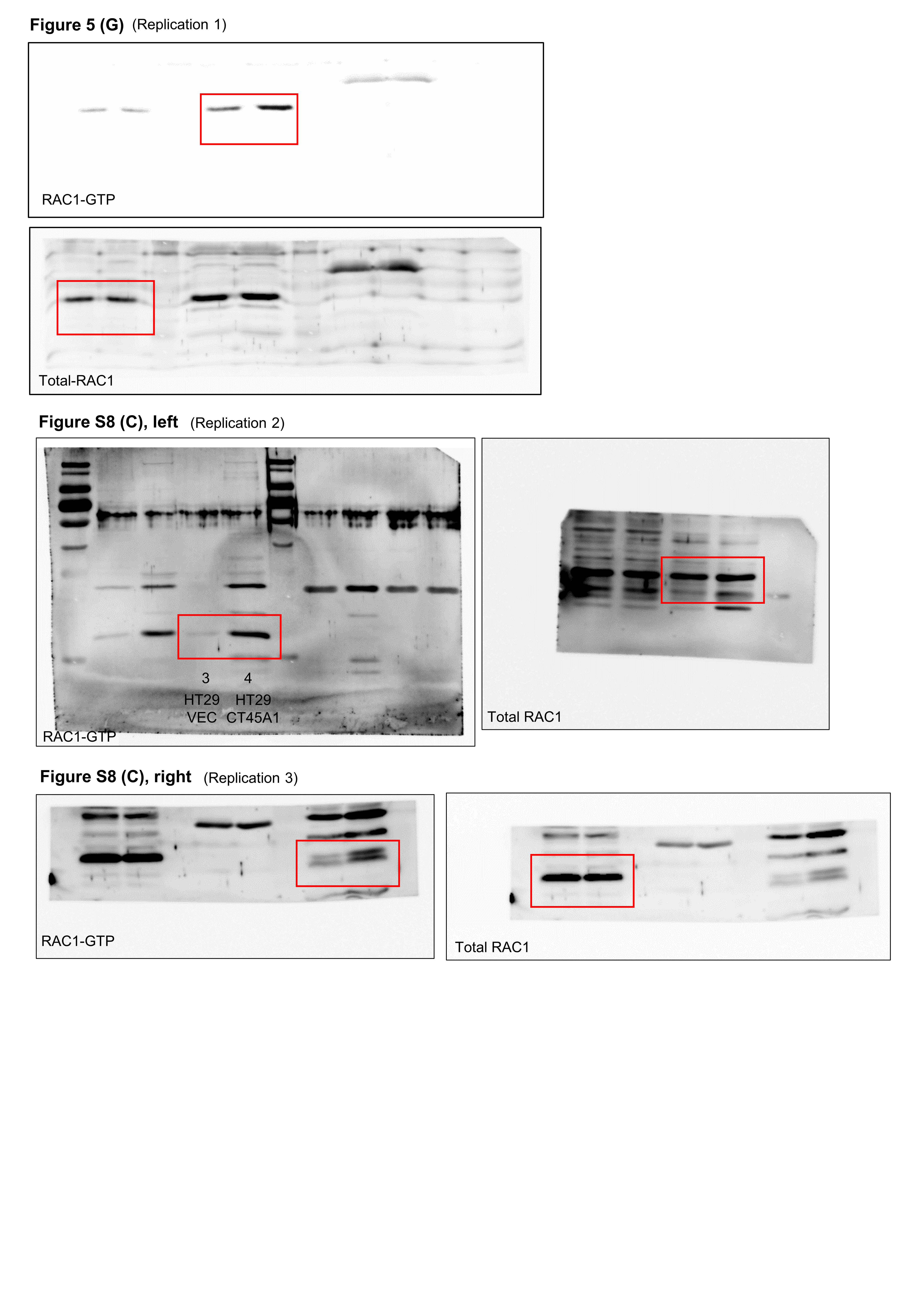

Supplement: Supplementary file 10 — Fig. S10. Uncropped western blot images. The uncropped blots and molecular weight labels are shown in the indicated images. [file MOL2-19-430-s012.zip › Figure S10-7.tif]
