# Supplementary material for: CT45A1‐mediated MLC2 (MYL9) phosphorylation promotes natural killer cell resistance and outer cell fate in a cell‐in‐cell structure, potentiating the progression of microsatellite instability‐high colorectal cancer
Source: Mol Oncol. 2024 Sep 25;19(2):430–51. doi: 10.1002/1878-0261.13736 (PMC11793002; doi:10.1002/1878-0261.13736)
Supplement: Supplementary file 11 — Table S1. Primer list for qPCR. [file MOL2-19-430-s013.docx]

| **Gene Symbol** | **Forward Primer** | **Reverse Primer** |
| --- | --- | --- |
| *ALCAM* | ACT TAC CAG GAC AGC CCG AA | ATG ACA CGG GTA GGG CAT CA |
| *ASCL2* | CAC ACA GGC TTC TCC CTA GC | CAA GAT CTG GAC ACG AGC AG |
| *CD44* | CCA GAT GGA GAA AGC TCT GA | GTC ATA CTG GGA GGT GTT TGG |
| *LGR5* | TGT TGG GAG ATC TGC TTT C | CAG ACG GTT TGA GGA AGA GA |
| *POU5F1* | ACC GAG TGA GAG GCA ACC | TGA GAA AGG AGA CCC AGC AG |
| *NANOG* | CAA CCA GAC CCA GAA CAT CC | TTC CAA AGC AGC CTC CAA G |
| *GAPDH* | AAG GTC GGA GTC AAC GGA TTT G | CCA TGG GTG GAA TCA TAT TGG AA |

**Supplementary Table 1: Primer list for qPCR.**
