# Supplementary material for: CT45A1‐mediated MLC2 (MYL9) phosphorylation promotes natural killer cell resistance and outer cell fate in a cell‐in‐cell structure, potentiating the progression of microsatellite instability‐high colorectal cancer
Source: Mol Oncol. 2024 Sep 25;19(2):430–51. doi: 10.1002/1878-0261.13736 (PMC11793002; doi:10.1002/1878-0261.13736)
Supplement: Supplementary file 12 — Table S2. Antibody list. [file MOL2-19-430-s015.docx]

**Supplementary Table 2: Antibody list.**

| **Antibody Name** | **Source** | **Identifier** | **Application** |
| --- | --- | --- | --- |
| Mouse APC-conjugated CD107a antibody | BioLegend | 328619 | FC (1:100) |
| Mouse APC-conjugated anti-PD-L1 antibody | BiolLegend | 393610 | FC (1:40) |
| Mouse Alexa Fluor 647 anti-human IgG Fc antibody | BioLegend | 409320 | FC (1:40) |
| Mouse Alexa Fluor 647 anti-HLA-A, B, C antibody | BioLegend | 311416 | FC (1:40) |
| Rabbit Alexa Fluor 647-conjugated anti-EPCAM antibody | ABclonal | A22486 | IFA (1:100) |
| Rabbit anti-CD47 antibody | ABclonal | A7278 | IFA (1:100) |
| Mouse Alexa Fluor 647-conjugated anti-EGFR antibody | BioLegend | 352918 | IFA (1:100) |
| Mouse Alexa APC anti-PD-L1 antibody | BioLegend | 393610 | IFA (1:100) |
| Goat Spark Red 718-conjugated anti-mouse IgG antibody | BioLegend | 405318 | IFA (1:100) |
| Alexa Fluor 647-conjugated Anti-rabbit IgG antibody | Cell Signaling | 4414S | IFA (1:100) |
| Mouse anti-phosphorylated MLC2 (S19) antibody | Cell Signaling | 3675S | IFA (1:100) |
| Rabbit anti-CT45A1antibody | OriGene | TA332257 | IFA (1:100)/ WB 1:1000) |
| Rabbit anti-CT45A1 antibody | Novus Biologicals | NBP2-46704 | IHC (1:200) |
| Mouse anti-phosphorylated MLC2 (S19) antibody | Cell Signaling | 3675S | IHC (1:50) |
| Goat IgG control antibody | R&D Systems | AB-108C | Neutralizing (100 μg/ml) |
| Mouse anti-PD-L1 antibody | Bioxcell | BE0285 | Neutralizing (100 μg/ml) |
| Rabbit anti-caspase-3 antibody | Cell Signaling | 9662 | WB (1:1000) |
| Rabbit anti-MLC2 antibody | Cell signaling | 3672S | WB (1:1000) |
| Rabbit anti-Rac1 antibody | Invitrogen | PA1-091 | WB (1:1000) |
| Mouse anti-FLAG M2 antibody | Sigma-Aldrich | F1804 | WB (1:1000) |
| Rabbit anti-Rho antibody | ThermoFisher | 1862332 | WB (1:1000) |
| Mouse anti-phosphorylated MLC2 (S19) antibody | Cell Signaling | 3675S | WB (1:500) |
| Rabbit anti-MYLK antibody | Proteintech | 21642-1-AP | WB (1:500) |
| Goat anti-mouse IgG-HRP | Genetex | GTX213111-01 | WB (1:5000) |
| Goat anti-rabbit IgG-HRP | Genetex | GTX26721 | WB (1:5000) |
| Mouse anti-β-actin antibody | Proteintech | 66009-1 | WB (1:5000) |

FC, flow cytometry; IFA, immunofluorescent assay; IHC, immunohistochemistry; WB, western blot
