# Supplementary material for: CT45A1‐mediated MLC2 (MYL9) phosphorylation promotes natural killer cell resistance and outer cell fate in a cell‐in‐cell structure, potentiating the progression of microsatellite instability‐high colorectal cancer
Source: Mol Oncol. 2024 Sep 25;19(2):430–51. doi: 10.1002/1878-0261.13736 (PMC11793002; doi:10.1002/1878-0261.13736)
Supplement: Supplementary file 13 — Table S3. List for the reagents and chemicals used in this study. [file MOL2-19-430-s004.docx]

**Supplementary Table 3: List for the reagents and chemicals used in this study.**

| **Reagents and chemicals** | **Source** | **Identifier** |
| --- | --- | --- |
| 2-Mercaptoethanol (2ME) | Gibco | 21985-023 |
| Antibody dilution buffer | Ventana | ADB250 |
| Anti-immunoglobulins | BioGenex | QP900-9LE (C) |
| BCA protein assay kit | ThermoFisher | 23227 |
| Bovine serum albumin (BSA) | Bioshop | ALB001 |
| Cell culture lysis 5x reagent | Promega | E153A |
| Chamber slide (4-well format) | ThermoFisher | 155383 |
| Chloroform | Honeywell | C2432 |
| Citric acid | Honeywell | 27109 |
| Crystal violet | Sigma-Aldrich | C0775 |
| DAB solution | Epredia | TA-060-QHDX |
| DAPI | Sigma-Aldrich | SI-F6057 |
| Dimethyl sulfoxide (DMSO) | Scharlau | SU01551000 |
| DMEM medium | Gibco | 11965-084 |
| dNTP | Bioman | D4100 |
| DTT | Bionovas | AM0670-DTT |
| Ethanol | Honeywell | 32221 |
| Ethylenediaminetetraacetic acid (EDTA) | J.T.Baker | 2589937 |
| Fetal bovine serum (FBS) | Gibco | 26140079 |
| Fetal bovine serum (FBS) | Hyclone | SH30084.03 |
| First-strand RT buffer (5x) | Bionovas | AM0670-5X |
| Folic acid | Sigma-Aldrich | F8758 |
| Glutathione (GSH) sepharose | Cytiva | 17513201 |
| Horse serum | Gibco | 16050-122 |
| Inositol | Sigma-Aldrich | I7508 |
| Isopropanol | Sigma-Aldrich | I9516 |
| Isopropyl β-D-1-thiogalactopyranoside (IPTG) | Sigma-Aldrich | 16758 |
| Kaiser's glycerol gelatin mounting medium | Millipore | 1.09242.0100 |
| L15 medium | Gibco | 11415-064 |
| LeGO-C2 (mCherry) | Addgene | 27339 |
| LeGO-V2 (Venus) | Addgene | 27340 |
| Mayer's hemalum solution | Sigma-Aldrich | 1.09249.0500 |
| MEM alpha medium | Gibco | 12561-049 |
| ML-7 | Cayman Chemical | 11801 |
| Nuclease-free water | Invitrogen | 10977023 |
| Paraformaldehyde (PFA) | Sigma-Aldrich | P6148 |
| Penicillin/ streptomycin | Gibco | 15140-122 |
| Phosphatase inhibitor | ThermoFisher | P5726 |
| Phosphate-buffered saline (PBS) | Bioman | PBS101000 |
| pLenti-CT45A1 plasmid | Origene | RC214947L3 |
| pLenti-Vector plasmid | Origene | PS100092 |
| pMDLg/pRRE plasmid | Addgene | 12251 |
| Poly (2-hydroxyethyl methacrylate), polyhema | Sigma-Aldrich | P3932 |
| Polybrene | Sigma-Aldrich | H9268 |
| Primary human NK cells | Lonza Bioscience | 2W-501 |
| Protease inhibitor | ThermoFisher | 88666 |
| pRSV-Rev plasmid | Addgene | 12253 |
| Random primer | Genestar | CA0360-0001 |
| Recombinant-siglec7-Fc protein | R&D Systems | 1138-SL-050 |
| Recombinant-siglec9-Fc protein | R&D Systems | 1139-SL-050 |
| Reconbinant interleukin-2 (IL-2) | PeproTech | 200-02 |
| Reverse transcriptase | Bionovas | AM0670-1000 |
| RPMI 1640 medium | Gibco | 11875-085 |
| Streptavidin peroxidase | BioGenex | QP900-9LE (B) |
| SYBR green master mix | ThermoFisher | 4385712 |
| Thiazolyl blue tetrazolium bromide reagent | Sigma-Aldrich | M5655 |
| T-pro NTR III transfection reagent | T-pro biotechnology | JT97-N006M |
| Transwell inserts with 8 μm pores | Costar | 3422 |
| Triton X-100 | Sigma-Aldrich | 9002-93-1 |
| TRIzol | ThermoFisher | 15596018 |
| Y-27632 | Cell Signaling Technology | 13624S |
