# Supplementary material for: CT45A1‐mediated MLC2 (MYL9) phosphorylation promotes natural killer cell resistance and outer cell fate in a cell‐in‐cell structure, potentiating the progression of microsatellite instability‐high colorectal cancer
Source: Mol Oncol. 2024 Sep 25;19(2):430–51. doi: 10.1002/1878-0261.13736 (PMC11793002; doi:10.1002/1878-0261.13736)
Supplement: Supplementary file 14 — Table S4. The aggressive MSI‐H CRC signature. [file MOL2-19-430-s005.docx]

**Supplementary Table 4: The aggressive MSI-H CRC signature.**

| **Gene symbol** | **FC^#^ (log2)** | **FDR^#^** |
| --- | --- | --- |
| MAGEA9B | 9.78 | 2.30E-11 |
| CT45A1 | 8.77 | 9.94E-15 |
| MAGEA3 | 8.47 | 2.23E-10 |
| CTAG2 | 8.34 | 3.82E-08 |
| MAGEA6 | 6.65 | 0.005651829 |
| MYBPC1 | 6.08 | 4.48E-14 |
| GABRA3 | 5.79 | 2.19E-09 |
| MAGEC2 | 5.36 | 4.66E-05 |
| IGF2BP1 | 5.19 | 1.72E-10 |
| SFTPB | 5.05 | 4.80E-06 |
| GRM1 | 4.94 | 3.26E-12 |
| TTYH1 | 4.90 | 4.15E-15 |
| EYA1 | 4.82 | 3.06E-10 |
| SLC34A2 | 4.19 | 0.001985764 |
| SFTPC | 3.91 | 8.93E-05 |
| KRT81 | 3.89 | 2.04E-08 |
| ANKLE1 | 3.79 | 2.19E-09 |
| NEB | 3.77 | 3.86E-05 |
| NAPSA | 3.77 | 1.35E-06 |
| IL1RL1 | 3.72 | 1.09E-05 |
| SP8 | 3.45 | 0.007221205 |
| CLDN9 | 3.13 | 0.000716915 |
| FOXH1 | 3.02 | 5.50E-05 |
| C8orf84 | 2.84 | 0.008908407 |
| BDNF | 2.61 | 0.001622819 |
| HS3ST2 | 2.56 | 0.001682331 |
| APOC1 | 2.54 | 0.000401936 |
| C20orf103 | 2.40 | 0.000716915 |
| WNT10A | 2.39 | 0.000952877 |
| APOE | 2.37 | 0.004033168 |
| PCDHB11 | 2.18 | 0.008908407 |
| TPPP3 | 2.05 | 0.009958249 |
| TNFRSF18 | 1.68 | 0.005651829 |

^#^FC: fold changes; FDR: false discovery rate.
