# Supplementary material for: CT45A1‐mediated MLC2 (MYL9) phosphorylation promotes natural killer cell resistance and outer cell fate in a cell‐in‐cell structure, potentiating the progression of microsatellite instability‐high colorectal cancer
Source: Mol Oncol. 2024 Sep 25;19(2):430–51. doi: 10.1002/1878-0261.13736 (PMC11793002; doi:10.1002/1878-0261.13736)
Supplement: Supplementary file 15 — Table S5. Upregulated CT45A1 signature in MSI‐H CRC cells. [file MOL2-19-430-s002.docx]

**Supplementary Table 5: Upregulated CT45A1 signature in MSI-H CRC cells.**

| **Gene ID** | **Gene Symbol** | **Ratio (DLD-1-CT45A1/DLD-1-Vec)** | **Ratio (HCT15-CT45A1/HCT15-Vec)** |
| --- | --- | --- | --- |
| ENSG00000228836 | CT45A5 | 3132.777694 | 899778.21 |
| ENSG00000268940 | CT45A1 | 267904.43 | 274799.21 |
| ENSG00000278289 | CT45A6 | 1518.145841 | 247573.97 |
| ENSG00000271449 | CT45A2 | 175352.84 | 222490.88 |
| ENSG00000273696 | CT45A7 | 674312.29 | 207108.65 |
| ENSG00000270946 | CT45A9 | 1230.612482 | 200130.98 |
| ENSG00000269096 | CT45A3 | 660119.02 | 119286.41 |
| ENSG00000285625 | AC117378.1 | 144045.93 | 57.08443452 |
| ENSG00000274847 | MAFIP | 4.179094704 | 14.72151423 |
| ENSG00000272741 | AC069257.3 | 7639.56 | 5.111723265 |
| ENSG00000257950 | P2RX5-TAX1BP3 | 2.746339131 | 4.872515894 |
| ENSG00000256500 | AL139300.1 | 2.559275272 | 4.105788359 |
| ENSG00000188626 | GOLGA8M | 3.860286959 | 4.025360338 |
| ENSG00000269403 | AC008750.7 | 1.630188598 | 3.72242086 |
| ENSG00000267059 | AC005943.1 | 4.816049281 | 3.385067742 |
| ENSG00000101335 | MYL9 | 3.477096388 | 3.319458789 |
| ENSG00000219435 | CATSPERZ | 21.75707426 | 3.001238146 |
| ENSG00000285920 | AC087721.2 | 1.699221629 | 2.910616136 |
| ENSG00000147576 | ADHFE1 | 1.74239905 | 2.722799094 |
| ENSG00000180767 | CHST13 | 2.524357548 | 2.7155727 |
| ENSG00000147437 | GNRH1 | 1.638227728 | 2.710909166 |
| ENSG00000166415 | WDR72 | 2.589389857 | 2.524415464 |
| ENSG00000286070 | AP000356.5 | 6.602330209 | 2.484925266 |
| ENSG00000102755 | FLT1 | 1.873458719 | 2.453961393 |
| ENSG00000166592 | RRAD | 3.051085429 | 2.42352135 |
| ENSG00000197889 | MEIG1 | 1.527810254 | 2.331290044 |
| ENSG00000173110 | HSPA6 | 1.976829955 | 2.325449302 |
| ENSG00000285130 | AL358113.1 | 4.125090193 | 2.265329215 |
| ENSG00000175449 | RFESD | 1.525119224 | 2.246951468 |
| ENSG00000161681 | SHANK1 | 9.056749432 | 2.225513257 |
| ENSG00000243566 | UPK3B | 1.630428717 | 2.191512785 |
| ENSG00000204428 | LY6G5C | 1.502424587 | 2.184933791 |
| ENSG00000127588 | GNG13 | 1.824097256 | 2.06547742 |
| ENSG00000151366 | NDUFC2 | 1.761823526 | 1.925442007 |
| ENSG00000196866 | H2AC7 | 2.097420872 | 1.924153682 |
| ENSG00000108107 | RPL28 | 1.562423846 | 1.844407771 |
| ENSG00000284491 | THSD8 | 6.851402721 | 1.828920315 |
| ENSG00000108387 | SEPTIN4 | 1.639611207 | 1.777177073 |
| ENSG00000241962 | AC079447.1 | 2.500567076 | 1.743003966 |
| ENSG00000184709 | LRRC26 | 1.509558826 | 1.727324787 |
| ENSG00000143127 | ITGA10 | 1.946270057 | 1.721862914 |
| ENSG00000178226 | PRSS36 | 2.08695627 | 1.710642315 |
| ENSG00000166455 | C16orf46 | 1.690943961 | 1.691856022 |
| ENSG00000205704 | LINC00634 | 2.496592923 | 1.671233223 |
| ENSG00000133106 | EPSTI1 | 1.507555355 | 1.612170684 |
| ENSG00000214309 | MBLAC1 | 1.520164446 | 1.607265385 |
| ENSG00000110195 | FOLR1 | 1.903500017 | 1.570577026 |
| ENSG00000163467 | TSACC | 1.62985088 | 1.565362061 |
| ENSG00000128564 | VGF | 1.660897079 | 1.557766646 |
| ENSG00000128383 | APOBEC3A | 1.669848553 | 1.541356892 |
| ENSG00000143512 | HHIPL2 | 3.399608373 | 1.514644327 |
| ENSG00000166532 | RIMKLB | 2.343042716 | 1.510588097 |
| ENSG00000171786 | NHLH1 | 2.707890194 | 1.502727002 |
