# Supplementary material for: CT45A1‐mediated MLC2 (MYL9) phosphorylation promotes natural killer cell resistance and outer cell fate in a cell‐in‐cell structure, potentiating the progression of microsatellite instability‐high colorectal cancer
Source: Mol Oncol. 2024 Sep 25;19(2):430–51. doi: 10.1002/1878-0261.13736 (PMC11793002; doi:10.1002/1878-0261.13736)
Supplement: Supplementary file 16 — Table S6. Downregulated CT45A1 signature in MSI‐H CRC cells. [file MOL2-19-430-s009.docx]

**Supplementary Table 6: Downregulated CT45A1 signature in MSI-H CRC cells.**

| **Gene ID** | **Gene Symbol** | **Ratio (DLD-1-Vec/DLD-1-CT45A1)** | **Ratio (HCT15-Vec/HCT15-CT45A1)** |
| --- | --- | --- | --- |
| ENSG00000286185 | AC242842.3 | 2.114428924 | 30939.05 |
| ENSG00000257921 | AC025165.3 | 6572.76 | 21661.97 |
| ENSG00000255071 | SAA2-SAA4 | 49561.68 | 7.708484295 |
| ENSG00000204866 | IGFL2 | 1.508670563 | 3.610278082 |
| ENSG00000259112 | NDUFC2-KCTD14 | 1.928325549 | 3.304566374 |
| ENSG00000106069 | CHN2 | 1.571241857 | 2.649988168 |
| ENSG00000203546 | AL139353.1 | 2.177138898 | 2.51708488 |
| ENSG00000255639 | AC005833.1 | 2.419218596 | 2.459980439 |
| ENSG00000260729 | AC009690.1 | 4.168455867 | 2.322709138 |
| ENSG00000152154 | TMEM178A | 1.869441057 | 2.318764577 |
| ENSG00000254870 | ATP6V1G2-DDX39B | 4.559570939 | 2.297166406 |
| ENSG00000131018 | SYNE1 | 2.967933365 | 2.291501047 |
| ENSG00000213648 | SULT1A4 | 1.605044688 | 2.255451199 |
| ENSG00000115008 | IL1A | 1.602751251 | 2.241396601 |
| ENSG00000163618 | CADPS | 2.174799888 | 2.197647836 |
| ENSG00000163491 | NEK10 | 2.23772787 | 2.142744381 |
| ENSG00000069535 | MAOB | 1.518490271 | 2.099606402 |
| ENSG00000113296 | THBS4 | 2.222565061 | 2.043579231 |
| ENSG00000186204 | CYP4F12 | 1.607491976 | 1.90474928 |
| ENSG00000261832 | AC138894.1 | 2.287554999 | 1.891438891 |
| ENSG00000132437 | DDC | 1.528691236 | 1.867865013 |
| ENSG00000140932 | CMTM2 | 5121.33 | 1.834883199 |
| ENSG00000158486 | DNAH3 | 4.167225177 | 1.739160238 |
| ENSG00000128000 | ZNF780B | 1.587209688 | 1.733966455 |
| ENSG00000272410 | AC022384.1 | 6.232809502 | 1.650741231 |
| ENSG00000168743 | NPNT | 1.670828494 | 1.63501088 |
| ENSG00000265972 | TXNIP | 1.881387878 | 1.607496633 |
| ENSG00000196511 | TPK1 | 1.507614142 | 1.606345504 |
| ENSG00000168477 | TNXB | 1.973102315 | 1.582818372 |
| ENSG00000088726 | TMEM40 | 2.807712413 | 1.564502289 |
| ENSG00000074527 | NTN4 | 1.732415646 | 1.564000865 |
| ENSG00000169330 | MINAR1 | 1.658704433 | 1.555286163 |
| ENSG00000163220 | S100A9 | 5.082865997 | 1.538540847 |
| ENSG00000231989 | PPP1R2B | 1.828490773 | 1.538234583 |
| ENSG00000100739 | BDKRB1 | 1.831900946 | 1.524935765 |
| ENSG00000138378 | STAT4 | 1.699377435 | 1.520603028 |
| ENSG00000240038 | AMY2B | 2.25840742 | 1.518530059 |
| ENSG00000238243 | OR2W3 | 1.546646459 | 1.5131752 |
| ENSG00000125895 | TMEM74B | 2.633844865 | 1.501583592 |
